# Anti-inflammatory Polyketides from the Marine-Derived Fungus *Eutypella scoparia*

**DOI:** 10.3390/md20080486

**Published:** 2022-07-28

**Authors:** Ya-Hui Zhang, Hui-Fang Du, Wen-Bin Gao, Wan Li, Fei Cao, Chang-Yun Wang

**Affiliations:** 1 Key Laboratory of Marine Drugs, the Ministry of Education of China, School of Medicine and Pharmacy, Institute of Evolution & Marine Biodiversity, Ocean University of China, Qingdao 266003, China; 15689932652@163.com; 2 College of Pharmaceutical Sciences, Key Laboratory of Pharmaceutical Quality Control of Hebei Province, Key Laboratory of Medicinal Chemistry and Molecular Diagnostics of Education Ministry of China, Hebei University, Baoding 071002, China; dhf12031203@163.com (H.-F.D.); liwanjingmin@163.com (W.L.); 3 Laboratory for Marine Drugs and Bioproducts, Qingdao National Laboratory for Marine Science and Technology, Qingdao 266237, China; 4 College of Life Sciences, Cangzhou Normal University, Cangzhou 061000, China; wenbinxing@yeah.net

**Keywords:** marine-derived fungus, *Eutypella scoparia*, polyketide, absolute configuration, anti-inflammatory activity

## Abstract

Three new polyketides, eutyketides A and B (**1** and **2**) and cytosporin X (**3**), along with four known compounds (**4**–**7**), were obtained from the marine-derived fungus *Eutypella scoparia*. The planar structures of **1** and **2** were elucidated by extensive HRMS and 1D and 2D NMR analyses. Their relative configurations of C-13 and C-14 were determined with chemical conversions by introducing an acetonylidene group. The absolute configurations of **1**–**3** were determined by comparing their experimental electronic circular dichroism (ECD) data with their computed ECD results. All of the isolated compounds were tested for their anti-inflammatory activities on lipopolysaccharide-induced nitric oxide production in RAW 264.7 macrophages. Compounds **5** and **6** showed stronger anti-inflammatory activities than the other compounds, with the inhibition of 49.0% and 54.9% at a concentration of 50.0 µg/mL, respectively.

## 1. Introduction

*Eutypella* species, which are one genus of the ubiquitous fungi, are widely distributed in many extreme environments, including Antarctica, tropical forests, and marine organisms [1,2,3]. Chemical investigations of *Eutypella* species have resulted in diverse metabolites, including *γ*-lactones, benzopyrans, cysporins, terpenoids, and nitrogen-containing compounds [4,5]. Among them, many bioactive secondary metabolites were obtained, such as antibacterial scoparasin B [5], cytotoxic phenochalasin B [6], and antitumor diaporthein B [7]. The *Eutypella* genus has become an attractive target for discovering leading compounds due to its remarkable biological activity and novel complex structures. In recent years, a lot of work has been carried out on the isolation, total synthesis, pharmacological research, and drug development for the genus *Eutypella* [8,9,10].

**Figure 1:**
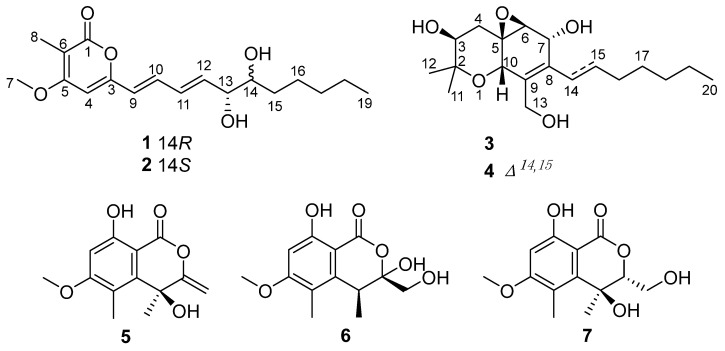
Chemical structures of compounds **1**−**7**.

As part of our ongoing investigation of bioactive natural products from marine-derived fungi [11,12,13,14,15,16], the strain *Eutypella scoparia* HBU-91 attracted our attention because the EtOAc extract of the culture showed anti-inflammatory activity. As a result, the new eutyketides A and B (**1** and **2**) and cytosporin X (**3**), together with four known compounds (**4**–**7**) (Figure 1), were obtained by using silica gel and LH-20 column chromatography and semipreparative HPLC. Structurally, compounds **1** and **2** were a pair of epimers with *vic*-diol unit on their side chain, while **3** exhibited a skeleton characterized by a polyketide moiety and a terpenoid part. All of the isolated compounds were tested for their anti-inflammatory activities. Herein, we report their isolation, structure elucidation, and biological activities.

## 2. Results and Discussion

### 2.1. Structural Elucidation

**Figure 2:**
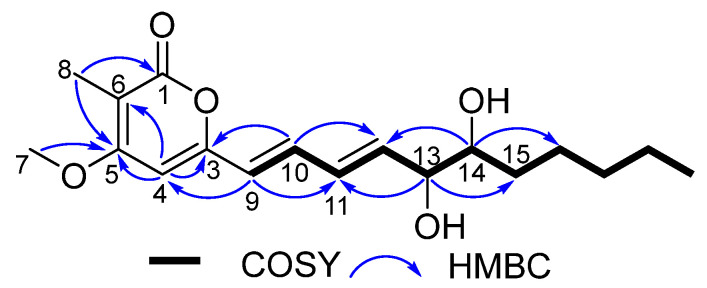
COSY and key HMBC correlations of compounds **1** and **2**.

Eutyketide A (**1**) was obtained as a pale yellow oil. The molecular formula of **1** was determined to be C_18_H_26_O_5_ based on the HRESIMS of the pseudomolecular ion (*m*/*z* 345.1669 [M + Na]^+^, calcd for C_18_H_26_O_5_Na, 345.1672), indicating six degrees of unsaturation. The IR spectrum suggested the presence of hydroxy (3385 cm^−1^), double bond (1558 cm^−1^), and ester carbonyl (1683 cm^−1^) functionalities. The ^1^H NMR spectrum (Table 1) showed resonances for five olefinic protons [*δ*_H_ 7.13 (dd, *J* = 15.0, 11.5 Hz), 6.42 (dd, *J* = 15.0, 11.5 Hz), 6.08 (d, *J* = 15.0 Hz), 6.07 (s), and 6.05 (dd, *J* = 15.0, 6.0 Hz)], two oxymethines [*δ*_H_ 4.23 (dd, *J* = 6.0, 4.2 Hz) and 3.72 (m)], a methoxy [*δ*_H_ 3.87 (s)], and two methyls [*δ*_H_ 1.93 (s) and 0.88 (t, *J* = 6.6 Hz)]. ^13^C NMR combined with HSQC spectra (Table 1) of **1** displayed 18 carbon resonances that could be assignable to 9 sp^2^ deshielded carbons, including a *α*,*β*-unsaturated carbonyl [*δ*_C_ 165.0, (C-1)] and 8 olefinic carbons [*δ*_C_ 157.3 (C-3), 96.1 (C-4), 165.8 (C-5), 103.2 (C-6), 122.8 (C-9), 134.8 (C-10), 130.8 (C-11), and 137.5 (C-12)], and 9 sp^3^ deshielded carbons, including a methoxy [*δ*_C_ 56.4, (C-7)], 2 oxymethines [*δ*_C_ 75.2 (C-13) and 74.6 (C-14)], 4 methylenes [*δ*_C_ 32.2 (C-15), 31.9 (C-17), 25.7 (C-16), and 22.7 (C-18)], and 2 methyls [*δ*_C_ 8.9 (C-8) and 14.1 (C-19)]. The ^1^H and ^13^C NMR data revealed that **1** shares the same carbon framework as graphostrin I, a polyketide obtained from the Atlantic hydrothermal fungus *Graphostroma* sp. MCCC 3A00421 [17]. The main differences between them were the presence of a methyl at C-6 and a *vic*-diol [−OHCH−CHOH−] substructure at C-13/14 in **1** instead of the group [−CH_2_−CH_2_−] and the absence of the hydroxy group at C-18 in graphostrin I. The above differences were confirmed by the COSY cross-peaks of H-12/H-13/H-14/H_2_-15 and H_2_-17/H_2_-18/H_3_-19 and the HMBC correlations from H-8 to C-1, C-5, and C-6, from H-13 to C-11 and C-15, and from H-14 to C-12 and C-16, respectively (Figure 2). In addition, *trans* geometries at C-9−C-10 and C-11−C-12 double bonds were assigned by the large coupling constants (*J*_9,10_ = 15.0 Hz and *J*_11,12_ = 15.0 Hz) [17]. By detailed analysis of its 2D NMR spectra, the planar structure of **1** was assigned.

Eutyketide B (**2**) was also obtained as a pale yellow oil. It exhibited the same molecular formula as **1**, C_18_H_26_O_5_, according to the pseudomolecular ion at *m*/*z* 345.1669 [M + Na]^+^ in the HRESIMS spectrum. Detailed analysis of the ^1^H and ^13^C NMR spectra of **2** (Table 1) revealed that its 1D NMR data were similar to those of **1**. The differences were attributable to the signals [*δ*_H_ 4.05 (m, H-13) and 3.51 (m, H-14); *δ*_C_ 138.7 (C-12), 74.4 (C-13), and 33.2 (C-15) in **2** vs. *δ*_H_ 4.23 (dd, *J* = 6.0, 4.2 Hz, H-13) and 3.72 (m, H-14); *δ*_C_ 137.5 (C-12), 75.2 (C-13), and 32.2 (C-15) in **1**], indicating that the structural differences between them should be located in this part of the structure (C-13 and C-14). Thus, it was deduced that **1** and **2** were either C-13 or C-14 epimers.

**Figure 3:**
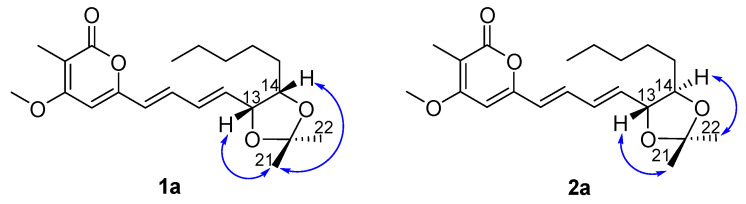
Structures and 1D NOE correlations of the acetonide products of **1a** and **2a**.

The structural differences between **1** and **2** and their relative configurations were elucidated on the basis of chemical conversions and 1D NOE experiments. Treatment of **1** and **2** with 2,2-dimethoxypropane in the presence of TsOH afforded **1a** and **2a** as the acetonide products. In the selective NOE of **1a** (Figure 3), irradiation of H-13 at *δ*_H_ 4.59 and H-14 at *δ*_H_ 4.17 resulted in the enhancement of H_3_-21, indicating that H-13 and H-14 should be placed on the same face of **1**. In the selective NOE of **2a** (Figure 3), irradiation of H-13 at *δ*_H_ 4.09 led to the enhancement of H_3_-21, while irradiation of H-14 at *δ*_H_ 3.70 caused the enhancement of H_3_-22, suggesting that H-13 and H-14 should be placed on the opposite side of **2**.

**Figure 4:**
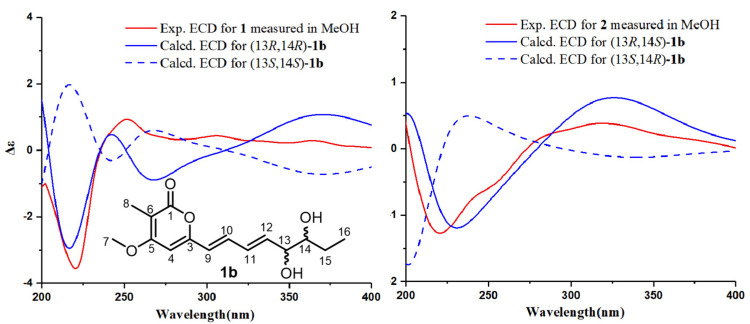
Calculated ECD spectra of (13*R*,14*R*)-**1b**, (13*S*,14*S*)-**1b**, (13*R*,14*S*)-**1b**, and (13*S*,14*R*)-**1b** and the experimental ECD spectra of **1** and **2**.

The calculation of the solution conformers is the most time-demanding part of the ECD calculation in conformationally flexible molecules and may be aided by simplifying the input geometry to reduce the number of conformers and save computational time [18]. For example, alkyl side chains and unsaturated side chains with isolated chromophores in an achiral environment could be simplified by truncation [18]. The absolute configuration of the hydroxyl group at C-13 was affected by the conjugate system, which can be determined by ECD calculation [19]. For **1** and **2**, the absolute configurations of C-13 and C-14 were determined by comparing their experimental electronic circular dichroism (ECD) results with the computed results of their simplified model compounds. The group of C-15 to C-19 was a saturated alkyl side chain with no chromophore and had a negligible effect on the ECD spectrum. Thus, the C-15 to C-19 alkyl substituent was truncated to a methyl group as model compound **1b** (Figure 4)**.** Molecules of (13*S*,14*S*)-**1b**, (13*R*,14*R*)-**1b**, (13*R*,14*S*)-**1b**, and (13*S*,14*R*)-**1b** were chosen for ECD calculations, which were carried out at the B3LYP/6-311+G(d,p) level in MeOH using the PCM model. The predicted ECD spectrum of (13*R*,14*R*)-**1b** matched well with the experimental ECD curve of **1**, and the predicted ECD spectrum of (13*R*,14*S*)-**1b** was in good agreement with the experimental ECD data of **2** (Figure 4). Therefore, the absolute configurations of **1** and **2** could be defined as 13*R*,14*R* and 13*R*,14*S*, respectively.

To the best of our knowledge, compounds **1** and **2** are very similar to prosolanapyrones and their congeners [20]. They share the same pyranone framework with long alkyl side chains. In addition to the conjugate double bonds, compounds **1** and **2** also contain a *vic*-diol unit on their side chains, while prosolanapyrones just possess double bonds on their side chains.

**Figure 5:**
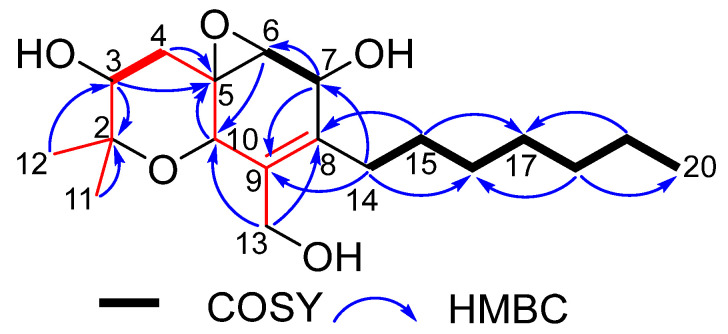
COSY and key HMBC correlations of **3**.

Cytosporin X (**3**) was obtained as a colorless oil. The molecular formula C_19_H_32_O_5_ was determined for **3** from the pseudomolecular ion peak at *m*/*z* 363.2132 [M + Na]^+^ (calcd 363.2142 for C_19_H_32_O_5_Na), which is consistent with four degrees of unsaturation. The IR spectrum of **3** at 3402 and 1652 cm^-1^ suggested the presence of hydroxyl and double bond groups. The ^1^H NMR spectrum of **3** displayed resonances for four oxygenated methine protons [*δ*_H_ 4.40 (s), 4.26 (d, *J* = 3.6 Hz), 3.67 (d, *J* = 12.0 Hz), and 3.24 (d, *J* = 3.6 Hz)], an oxygenated methylene proton [*δ*_H_ 4.24 (d, *J* = 12.0 Hz) and 4.04 (d, *J* = 12.0 Hz)], two singlet methyl protons [*δ*_H_ 1.32 (s) and 1.30 (s)], a terminal methyl proton [*δ*_H_ 0.87 (t, *J* = 6.6 Hz)], and a series of multiplet signals (Table 2). The ^13^C NMR spectrum of **3** revealed 19 resonances, including 2 olefinic carbons [*δ*_C_ 128.3 (C-8) and 138.1 (C-9)], 2 oxygenated quaternary carbons [*δ*_C_ 56.1 (C-5) and 77.2 (C-2)], 4 oxygenated methine carbons [*δ*_C_ 60.1 (C-6), 67.2 (C-7), 68.6 (C-10), and 73.5 (C-3)], an oxygenated methylene carbon [*δ*_C_ 62.2 (C-13)], 7 methylene carbons [*δ*_C_ 22.7 (C-19), 29.0 (C-15), 29.2 (C-17), 29.8 (C-16), 30.6 (C-14), 31.9 (C-18), and 35.6 (C-4)], and 3 methy carbons [*δ*_C_ 28.0 (C-12), 16.3 (C-11), and 14.2 (C-20)] (Table 2). The NMR data revealed that **3** belongs to the family of hexahydrobenzopyrane skeletons and is characterized by a polyketide moiety and a terpenoid part (the red part in Figure 5) with a tricyclic structure containing a hexahydrobenzopyrane moiety fused with an oxirane ring [1]. Careful comparison of the NMR data of **3** with those of the known hexahydrobenzopyrane cytosporin D (**4**) indicated that the structure of **3** is closely related to **4**. The notable difference between them lay in the presence of two methylene signals [*δ*_H_ 2.27 and 2.16 (H-14), 1.33 and 1.42 (H-15); *δ*_C_ 30.6 (C-14) and 29.0 (C-15) in **3**] and the absence of two olefinic methine signals [*δ*_H_ 6.48 (H-14), 6.15 (H-15); *δ*_C_ 124.8 (C-14) and 135.9 (C-15) in **4]**. The COSY cross-peaks of H-14/15/16 and the key HMBC correlations from H-14 to C-7/C-9 and from H-15 to C-8/C-17 (Figure 5) confirmed the above difference. Therefore, the planar structure of **3** was established.

**Figure 6:**
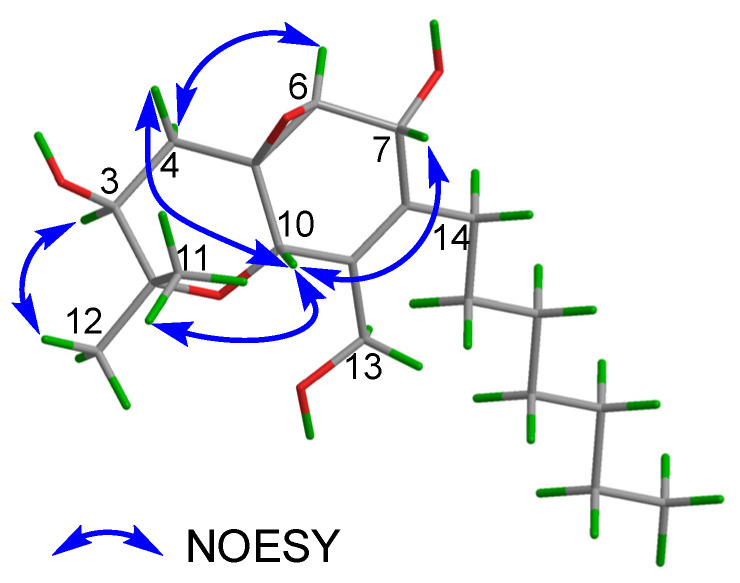
Key NOESY correlations of **3**.

The relative configuration of **3** was determined by analysis of the NOESY data (Figure 6). The NOESY correlations of H-3/H_3_-12, H_3_-11/H-10, H-10/H-7, H-10/H-4*β*, and H-4*α*/H-6 indicated that H-3 and H-6 were situated on the same side of the molecule with an *α*-orientation, while C-5, C-6, H-7, and H-10 were accordingly assigned to be *β*-configured. In addition, the observed NOEs are consistent with the structure and relative configuration of **4**.

**Figure 7:**
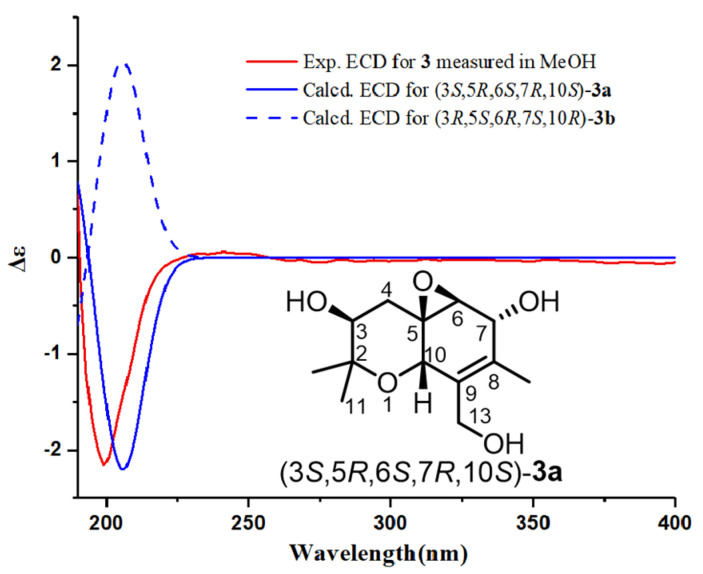
Calculated ECD spectra of (3*S*,5*R*,6*S*,7*R*,10*S*)-**3a** and (3*R*,5*S*,6*R*,7*S*,10*R*)-**3a** and the experimental ECD spectrum of **3**.

The absolute configuration of **3** was determined on the basis of ECD calculations. Compound **3** had a long flexible side chain with no chromophore. Thus, the side chain was truncated to two methyl groups attached at C-8, and model compound **3a** was used for ECD calculations. The calculations were carried out for (3*S*,5*R*,6*S*,7*R*,10*S*)-**3a** and (3*R*,5*S*,6*R*,7*S*,10*R*)-**3a** at the B3LYP/6-311+G(d,p) level using the PCM model (MeOH). The calculated ECD curve of (3*S*,5*R*,6*S*,7*R*,10*S*)-**3a** matched well with the experimental ECD data of **3** (Figure 7). Therefore, the absolute configuration of **3** was defined as 3*S*,5*R*,6*S*,7*R*,10*S*.

The known compounds **4**−**7** were identified as cytosporin D (**4**) [1], 4,8-dihydroxy-6-methoxy-4,5-dimethyl-3-methyleneisochroman-1-one (**5**) [21], banksialactone A (**6**) [22], and 4,8-dihydroxy-3-(hydr-oxymethyl)-6-methoxy-4,5-dimethylisochroman-1-one (**7**) [23], respectively, by comparing their NMR and MS data with reported values.

### 2.2. Anti-Inflammatory Activity

The anti-inflammatory activities of **1**−**7** were tested by evaluating their influence on nitric oxide (NO) production in RAW264.7 cells induced by lipopolysaccharide (LPS). Compounds **5** and **6** showed stronger anti-inflammatory activities than other compounds, with inhibition rates of 49.0%, 32.1%, and 27.4% for **5** and 54.9%, 35.9%, and 21.1% for **6** at concentrations of 50.0, 25.0, and 12.5 µg/mL, respectively. Moreover, **5** was also active at 6.25 µg/mL with 24.1% inhibition. In addition, **1** exhibited 20.3% inhibition when tested at 6.25 µg/mL (Table S2).

## 3. Materials and Methods

### 3.1. General Experimental Procedures

The OR data were recorded on a JASCO P-2000 spectrometer (Jasco Corp., Tokyo, Japan) in MeOH. ECD and UV spectra were measured by MOS450-SFM300 (Biologic, Grenoble, France) and a Perkin-Elmer model 241 spectrophotometers (Perkin-Elmer Corp., Waltham, MA, USA), respectively, with samples dissolved in MeOH. IR spectra were acquired on an FTIR-8400 spectrometer (Shimadzu, Kyoto, Japan) using KBr pellets. NMR data were recorded on a Bruker AV-600 spectrometer (Bruker Corp., Rheinstetten, Germany) with TMS as the internal standard. HRESIMS spectra were obtained from a Bruker apex-ultra 7.0T spectrometer (Bruker Corp., Rheinstetten, Germany). HPLC separation was performed on the Shimadzu LC-20AT system (Shimadzu, Kyoto, Japan) using an RP-18 HPLC column (Waters, Worcester, MA, USA, 10 × 250 mm, 5 μm).

### 3.2. Isolation of Fungal Material

#### 3.2.1. Fungal Material

The fungal strain *Eutypella scoparia* HBU-91 (GenBank, OM892669) was collected from the Bohai Sea (Huanghua, China, Apr. 2017). The strain was deposited in the College of Pharmaceutical Sciences, Hebei University, Baoding, China.

#### 3.2.2. Fermentation and Purification

Fermentation was carried out for the fungus *E. scoparia* using rice medium (170 mL water and 200 g rice in 1 L Erlenmeyer flasks, 200 flasks) at 28 °C for 40 days. After cultivation, the fermented rice substrate was extracted with a mixture of CH_2_Cl_2_/MeOH (1:1, 500 mL for each flask) five times and EtOAc five times successively to produce a residue (40.0 g), which was further subjected to silica gel column chromatography (CC), eluting with EtOAc−petroleum ether (PE) stepped gradient elution (0%–100%), to afford six fractions, Fr.1−Fr.6. Fr.2 was separated by Sephadex LH-20 CC (PE−CH_2_Cl_2_−MeOH (*v*/*v*, 2:1:1)) to afford four subfractions, Fr.2-1−Fr.2-4. Then, Fr.2-2 was fractionated by silica gel CC (PE−EtOAc, 5:1) and further purified by semipreparative HPLC (MeOH−H_2_O, 70:30, 2.0 mL/min) to afford **6** (2.3 mg) and **5** (3.9 mg). Fr.4 was separated by Sephadex LH-20 CC (MeOH−CH_2_Cl_2_ (*v*/*v*, 1:1)) to give subfractions Fr.4-1−Fr.4-3. Fr.4-1 was fractionated by silica gel CC (PE−Acetone, 3:1) and further purified by semipreparative HPLC (MeOH−H_2_O, 70:30, 2.0 mL/min) to give **4** (15.9 mg). Compound **3** (15.5 mg) was obtained from Fr.4-2 under the same conditions as **4.** Fr.4-3 was fractionated by semipreparative HPLC (MeOH−H_2_O, 60:40, 2 mL/min) to provide **1** (4.0 mg) and **2** (3.2 mg). Fr.5 was separated by Sephadex LH-20 CC (MeOH−CH_2_Cl_2_ (*v*/*v*, 1:1)) to give subfractions Fr.5-1 and Fr.5-2. Fr.5-2 was fractionated by silica gel CC (CH_2_Cl_2_/MeOH, 80:1) and further purified by semipreparative HPLC (MeOH−H_2_O, 50:50, 2.0 mL/min) to afford **7** (4.8 mg).

Eutylactone A (**1**): pale yellow oil; ${\left[\alpha \right]}_{20}^{D}$ +105.8 (*c* 0.1, MeOH); UV (MeOH) *λ*_max_ (log *ε*) 225 (2.85), 396 (2.36) nm; ECD (1.04 mM, MeOH) *λ*_max_ (Δ*ε*) 221 (−3.5), 251 (+0.9) nm; IR (KBr) *v*_max_ 3385, 2932, 2341, 1683, 1558, 1027 cm^−1^; ^1^H and ^13^C NMR data see Table 1; HRESIMS *m*/*z* 345.1669 [M + Na]^+^ (calcd for C_18_H_26_O_5_Na, 345.1672 [M + Na]^+^).

Eutylactone B (**2**); pale yellow oil; ${\left[\alpha \right]}_{20}^{D}$ +9.5 (*c* 0.1, MeOH); UV (MeOH) *λ*_max_ (log *ε*) 223 (2.85), 392 (2.39) nm; ECD (1.04 mM, MeOH) *λ*_max_ (Δ*ε*) 221 (−1.3), 319 (+0.4) nm; IR (KBr) *v*_max_ 3300, 2929, 2359,1679, 1556, 1027 cm^−1^; ^1^H and ^13^C NMR data see Table 1; HRESIMS *m*/*z* 345.1669 [M + Na]^+^ (calcd for C_18_H_26_O_5_Na, 345.1672 [M + Na]^+^).

Cytosporin X (**3**); pale yellow oil; ${\left[\alpha \right]}_{20}^{D}$ −314.5 (*c* 0.1, MeOH); UV (MeOH) *λ*_max_ (log *ε*) 254 (2.64), 335 (2.04) nm; ECD (0.98 mM, MeOH) *λ*_max_ (Δ*ε*) 199 (−2.15) nm; IR (KBr) *v*_max_ 3402, 2918, 2351, 1652, 1018 cm^−1^; ^1^H and ^13^C NMR data see Table 2; HRESIMS *m*/*z* 363.2132 [M + Na]^+^ (calcd for C_19_H_32_O_5_Na, 363.2142 [M + Na]^+^); 379.1871 [M + K]^+^ (calcd for C_19_H_32_O_5_K, 379.1881 [M + K]^+^).

#### 3.2.3. Acetonide Formation of **1** and **2**

A mixture of **1** (1.0 mg), 2,2-dimethoxypropane (2.0 mL), and *p*-TsOH (0.2 mg) was stirred at room temperature for 0.5 h. Saturated aqueous NaHCO_3_ (6.0 mL) was then added, and the reaction mixture was extracted with EtOAc (24 mL × 3). The organic solvents were removed with a high-vacuum pump, and the crude mixture was subjected to preparative HPLC to obtain acetonide product **1a** (0.96 mg). Acetonide product **2a** (0.91 mg) was obtained from **2** under the same conditions as **1a.**

Compound **1a**: ^1^H NMR (600 MHz, CDCl_3_) *δ* 7.17 (1H, dd, *J* = 15.1, 10.9 Hz, H-10), 6.74 (1H, dd, *J* = 15.0, 5.6 Hz, H-11), 6.37 (1H, dd, *J* = 15.0, 10.9 Hz, H-9), 6.06 (1H, s, H-4), 5.96 (1H, dd, *J* = 15.0, 7.2 Hz, H-12), 4.59 (1H, m, H-13), 4.35 (1H, s, H-14), 3.88 (3H, m, H-7), 1.94 (3H, s, H-8), 1.38 (3H, s, H-21), 1.34 (3H, s, H-22), 1.30-1.50 (8H, m, H-15/16/17/18), 0.88 (3H, t, *J* = 6.6 Hz, H-19).

Compound **2a**: ^1^H NMR (600 MHz, CDCl_3_) *δ* 7.15 (1H, dd, *J* = 15.0, 11.1 Hz, H-10), 6.43 (1H, dd, *J* = 15.0, 11.1 Hz, H-11), 6.09 (1H, d, *J* = 15.0 Hz, H-9), 6.06 (1H, s, H-4), 5.94 (1H, dd, *J* = 15.0, 7.2 Hz, H-12), 4.09 (1H, m, H-13), 3.88 (3H, s, H-8), 3.70 (1H, m, H-14), 1.95 (3H, s, H-8), 1.26 (3H, s, H-21), 1.25 (3H, s, H-22), 1.30-1.50 (8H, m, H-15/16/17/18), 0.89 (3H, t, *J* = 6.6 Hz, H-19).

### 3.3. Computational Section

A conformational search for the molecules was carried out using the MMFF94S force field and Compute VOA software, with relative energies ranging from 0−10.0 kcal/mol energy, respectively. The conformers were optimized at the B3LYP/6-31G(d)//B3LYP/6-311+G(d) levels with Gaussian 09 software [24]. Then, stable conformers with relative energy within a 2.5 kcal/mol energy window were chosen for ECD calculations at the B3LYP/6-311+G(d,p) level in methanol using the PCM model, with a total of 60 excited states. A standard deviation of 0.3 eV was used for ECD simulations. Boltzmann statistics were applied for the final simulations of the ECD spectra by the software SpecDis 1.64 [25].

### 3.4. Cell Culture and Viability Assay

Compounds **1**−**7** were first tested for their cytotoxic effects on RAW264.7 cells at 3.13, 6.25, 12.5, 25.0, and 50.0 µg/mL. Murine monocytic RAW264.7 macrophages were cultivated at 37 °C with 5% CO_2_ in Dulbecco’s modified Eagle’s medium (DMEM), which was added with 10% (*v*/*v*) fetal bovine serum (FBS) as well as 1% (*v*/*v*) penicillin/streptomycin. RAW264.7 cells were grown in 96-well plates and then incubated with the tested compounds for 24 h. A solution of 3-(4,5-dimethylthiazol-2-yl)-2,5-diphenyl tetrazolium bromide (MTT) at a concentration of 5.0 mg/mL was substituted for the culture medium. After incubation at 37 °C for 4 h, the MTT solution was removed, and DMSO was chosen for the dissolution of the formazan crystals. The absorbance was measured at 540 nm with a microplate reader [26]. Compounds **3**−**7** exhibited no toxicity at a concentration of 50.0 µg/mL, **1** showed no toxicity at a concentration of 25.0 µg/mL, and **2** displayed no toxicity at a concentration of 6.25 µg/mL.

### 3.5. Inhibition of NO Production Assay

The Griess assay was applied to evaluate the production of NO through the level of nitrite (NO_2_) in the medium [27]. RAW264.7 cells were inoculated into 96-well plates, and then LPS at a concentration of 1.0 μg/mL was added to induce inflammation. The tested compounds at different concentrations were added to the above mixture. The Griess reaction was used for the quantification of NO production in the supernatant. The absorbance was measured at 540 nm with a microplate reader. All of the experiments were carried out in triplicate.

## 4. Conclusions

In summary, three new polyketides (**1**−**3**), together with four known compounds (**4**−**7**), were isolated from the marine-derived fungus *Eutypella scoparia*. Chemical conversions and TDDFT ECD calculations were used to determine the absolute configurations of **1**−**3**. Compounds **1**, **5**, and **6** exhibited certain anti-inflammatory activities on nitric oxide (NO) production in RAW264.7 cells induced by lipopolysaccharide (LPS). Our findings will contribute to the diversity of these fungal metabolites.

## Figures and Tables

**Table 1:** ^1^H (600 MHz) and ^13^C (150 MHz) NMR Data of **1** and **2** in CDCl_3_.

No.	1		2	
	*δ*_C_, Type	*δ*_H_ (*J* in Hz)	*δ*_C_, Type	*δ*_H_ (*J* in Hz)
1	165.0, C	-	165.0, C	-
3	157.3, C	-	157.2, C	-
4	96.1, CH	6.07, s	96.2, CH	6.06, s
5	165.8, C	-	165.8, C	-
6	103.2, C	-	103.3, C	-
7	56.4, CH_3_	3.87, s	56.4, CH_3_	3.87, s
8	8.9, CH_3_	1.93, s	9.0, CH_3_	1.93, s
9	122.8, CH	6.08, d (15.0)	123.0, CH	6.07, d (15.2)
10	134.8, CH	7.13, dd (15.0, 11.5)	134.7, CH	7.13, dd (15.2, 11.1)
11	130.8, CH	6.42, dd (15.0, 11.5)	130.7, CH	6.44, dd (15.2, 11.1)
12	137.5, CH	6.05, dd (15.0, 6.0)	138.7, CH	6.01, dd (15.2, 6.2)
13	75.2, CH	4.23, dd (6.0, 4.2)	74.4, CH	4.05, m
14	74.6, CH	3.72, m	74.7, CH	3.51, m
15	32.2, CH_2_	1.42, m	33.2, CH_2_	1.47, m
16	25.7, CH_2_	1.31, m; 1.50, m	25.4, CH_2_	1.48, m
17	31.9, CH_2_	1.29, m	31.9, CH_2_	1.30, m
18	22.7, CH_2_	1.30, m	22.7, CH_2_	1.29, m
19	14.1, CH_3_	0.88, t (6.6)	14.1, CH_3_	0.88, t (6.6)

**Table 2:** ^1^H (600 MHz) and ^13^C (150 MHz) NMR Data of **3** in CDCl_3_.

No.	*δ*_C_, Type	*δ*_H_ (*J* in Hz)
2	77.2, C	-
3	73.5, CH	3.67, d (12.0)
4	35.6, CH_2_	2.23, dd (13.2, 5.4); 1.67, dd (13.2, 5.4)
5	56.1, C	-
6	60.1, CH	3.24, d (3.6)
7	67.2, CH	4.26, d (3.6)
8	128.3, C	-
9	138.1, C	-
10	68.6, CH	4.40, s
11	16.3, CH_3_	1.32, s
12	28.0, CH_3_	1.30, s
13	62.2, CH_2_	4.24, d (12.0); 4.04, d (12.0)
14	30.6, CH_2_	2.27, m; 2.16, m
15	29.0, CH_2_	1.33, m; 1.42, m
16	29.8, CH_2_	1.25, m
17	29.2, CH_2_	1.26, m
18	31.9, CH_2_	1.24, m
19	22.7, CH_2_	1.27, m
20	14.2, CH_3_	0.87, t (6.6)

## Data Availability

Data are contained within the article or Supplementary Material.

## Supplementary Materials

The following supporting information can be downloaded at: https://www.mdpi.com/article/10.3390/md20080486/s1. Figures S1−S29: 1D and 2D NMR, HRESIMS, and IR and UV spectra of compounds **1**−**3** and 1D NOE of **1a** and **2a**; Table S1: Cytotoxic activity data of compounds **1**−**7**; Table S2: Anti-inflammatory activity data of compounds **1**−**7**; Tables S3−S8: The coordinates for the lowest-energy conformers of **1b** and **3a** in ECD calculations.
