# Supplementary material for: Anti-inflammatory Polyketides from the Marine-Derived Fungus Eutypella scoparia
Source: Mar Drugs. 2022 Jul 28;20(8):486. doi: 10.3390/md20080486 (PMC9410037; doi:10.3390/md20080486)
Supplement: Supplementary file 1 [file marinedrugs-20-00486-s001.zip › marinedrugs-1832481-supplementary.pdf]

Anti-inflammatory Polyketides from the Marine-Derived Fungus *Eutypella scoparia*

Ya-Hui Zhang [1,2,3], Hui-Fang Du [2], Wen-Bin Gao [4], Wan Li [2], Fei Cao [2,*], and Chang-Yun Wang [1,3,*]

[1] Key Laboratory of Marine Drugs, the Ministry of Education of China, School of Medicine and Pharmacy; Institute of Evolution & Marine Biodiversity, Ocean University of China, Qingdao 266003, China; 15689932652@163.com (Y.-H.Z); changyun@ouc.edu.cn (C.-Y.W.)
[2] College of Pharmaceutical Sciences, Key Laboratory of Pharmaceutical Quality Control of Hebei Province, Key Laboratory of Medicinal Chemistry and Molecular Diagnostics of Education Ministry of China, Hebei University, Baoding 071002, China; dhf12031203@163.com (H.-F.D.); liwanjingmin@163.com (W.L.); caofei542927001@163.com (F.C.)
[3] Laboratory for Marine Drugs and Bioproducts, Qingdao National Laboratory for Marine Science and Technology, Qingdao 266237, People's Republic of China
[4] College of Life Sciences, Cangzhou Normal University, Cangzhou 061000, China; wenbinxing@yeah.net (W.-B.G.)
* Correspondence: changyun@ouc.edu.cn (C.-Y.W.); caofei542927001@163.com (F.C.)

List of Supporting Information

Figure S1. ^{1}H NMR (600 MHz, $CDCl_3$) spectrum of compound **1**.

Figure S2. ^{13}C NMR (150 MHz, $CDCl_3$) spectrum of compound **1**.

Figure S3. HSQC ($CDCl_3$) spectrum of compound **1**.

Figure S4. ^{1}H–^{1}H COSY ($CDCl_3$) spectrum of compound **1**.

Figure S5. HMBC ($CDCl_3$) spectrum of compound **1**.

Figure S6. HRESIMS spectrum of compound **1**.

Figure S7. IR spectrum of compound **1**.

Figure S8. Experimental UV spectrum of **1**.

Figure S9. ^{1}H NMR and 1D NOE (resonated at H-13) spectra of compound **1a**.

Figure S10. ^{1}H NMR and 1D NOE (resonated at H-14) spectra of compound **1a**.

Figure S11. ^{1}H NMR (600 MHz, $CDCl_3$) spectrum of compound **2**.

Figure S12. ^{13}C NMR (150 MHz, $CDCl_3$) spectrum of compound **2**.

Figure S13. HSQC ($CDCl_3$) spectrum of compound **2**.

Figure S14. ^{1}H–^{1}H COSY ($CDCl_3$) spectrum of compound **2**.

Figure S15. HMBC ($CDCl_3$) spectrum of compound **2**.

Figure S16. HRESIMS spectrum of compound **2**.
Figure S17. IR spectrum of compound **2**.
Figure S18. Experimental UV spectrum of **2**
Figure S19. ^{1}H NMR and 1D NOE (resonated at H-13) spectra of compound **2a**.
Figure S20. ^{1}H NMR and 1D NOE (resonated at H-14) spectra of compound **2a**.
Figure S21. ^{1}H NMR (600 MHz, $CDCl_3$) spectrum of compound **3**.
Figure S22. ^{13}C NMR (150 MHz, $CDCl_3$) spectrum of compound **3**.
Figure S23. HSQC ($CDCl_3$) spectrum of compound **3**.
Figure S24. ^{1}H–^{1}H COSY ($CDCl_3$) spectrum of compound **3**.
Figure S25. HMBC ($CDCl_3$) spectrum of compound **3**.
Figure S26. NOESY ($CDCl_3$) spectrum of compound **3**.
Figure S27. HRESIMS spectrum of compound **3**.
Figure S28. IR spectrum of compound **3**.
Figure S29. Experimental UV spectrum of **3**.

Table S1. Cytotoxic activity data of compounds **1**−**7**.

Table S2. Anti-inflammatory activity data of compounds **1**−**7**.

Table S3. The coordinate for the lowest-energy conformer of compound (13*S*,14*S*)-**1b** in ECD calculation
Table S4. The coordinate for the lowest-energy conformer of compound (13*R*,14*R*)-**1b** in ECD calculation
Table S5. The coordinate for the lowest-energy conformer of compound (13*R*,14*S*)-**1b** in ECD calculation
Table S6. The coordinate for the lowest-energy conformer of compound (13*S*,14*R*)-**1b** in ECD calculation
Table S7. The coordinate for the lowest-energy conformer of compound (3*S*,5*R*,6*S*,7*R*,10*S*)-**3a** for ECD calculation
Table S8. The coordinate for the lowest-energy conformer of compound (3*R*,5*S*,6*R*,7*S*,10*R*)-**3a** for ECD calculation

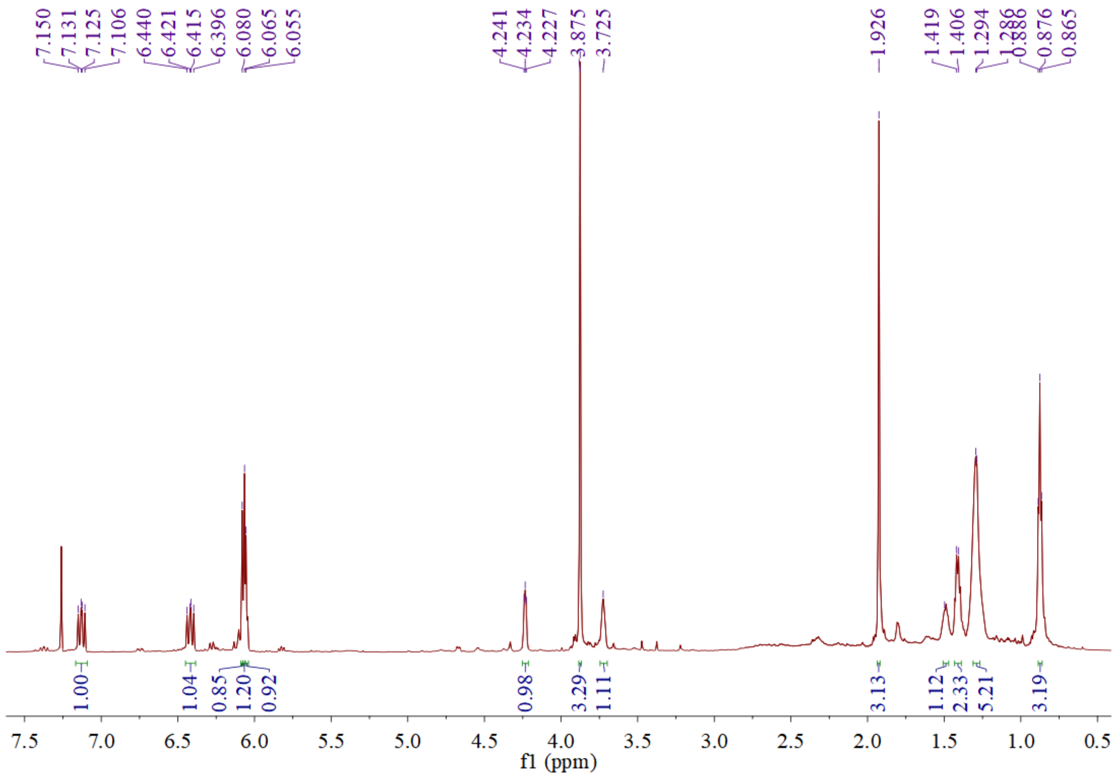

Figure S1. ^{1}H NMR (600 MHz, $CDCl_3$) spectrum of compound **1**.

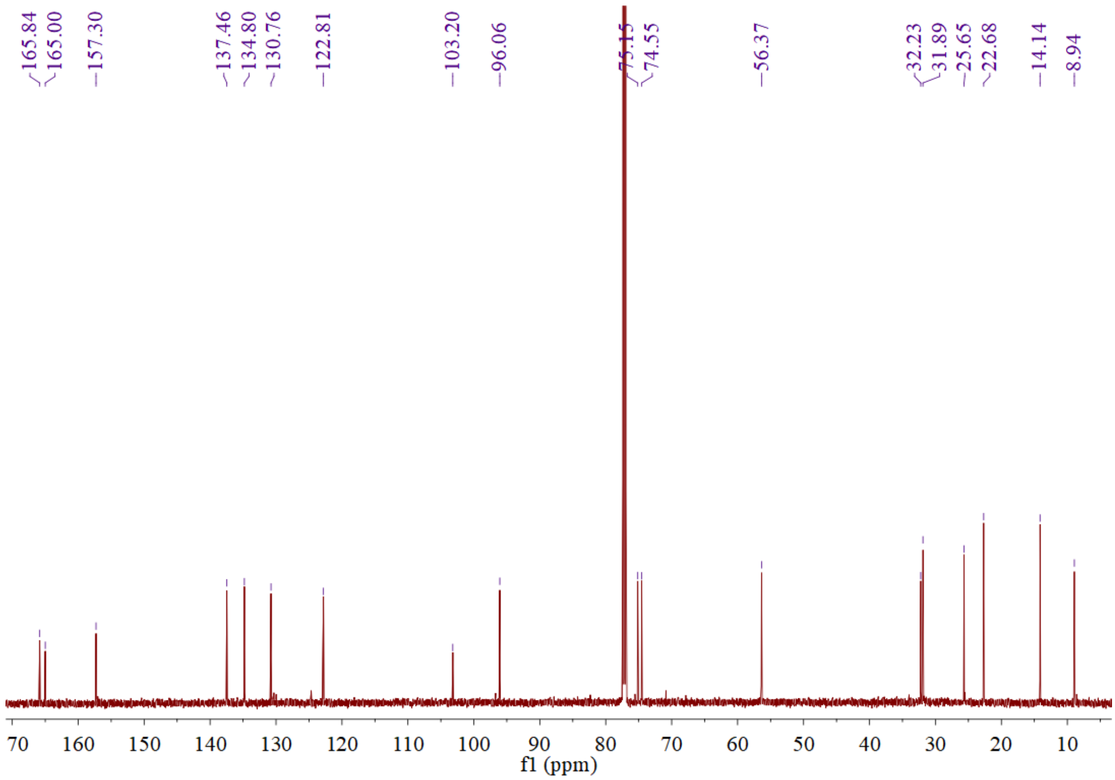

Figure S2. ^{13}C NMR (150 MHz, $CDCl_3$) spectrum of compound **1**.

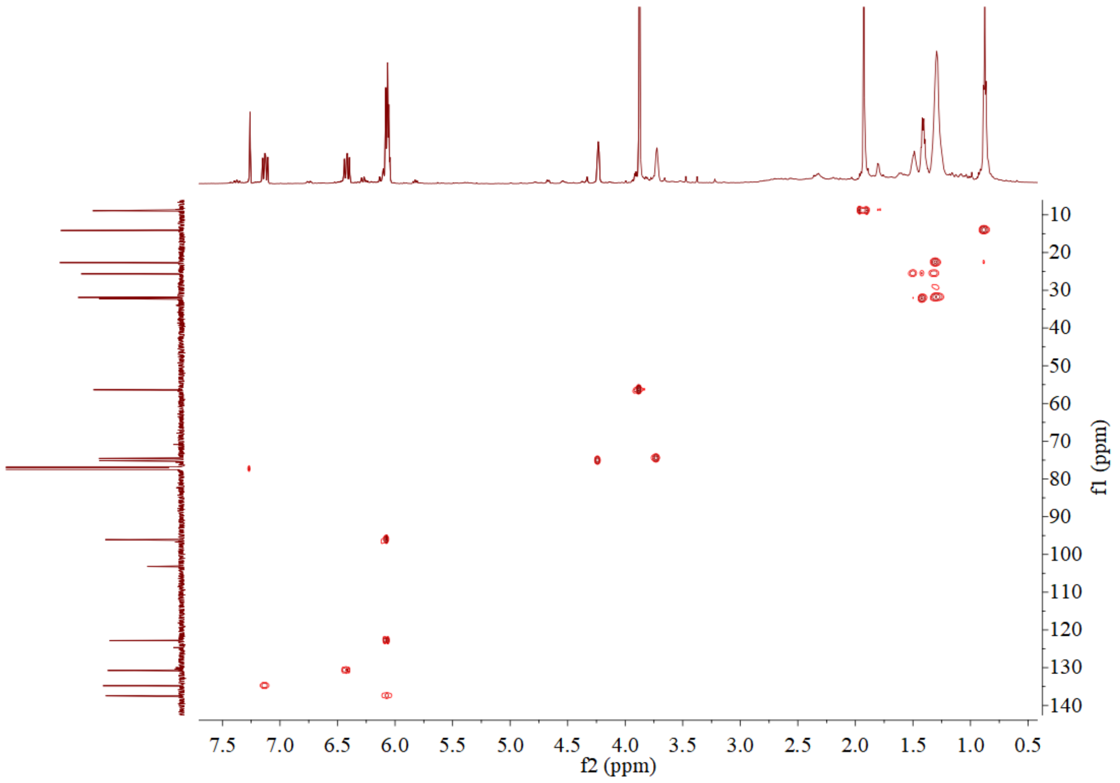

Figure S3. HSQC ($CDCl_3$) spectrum of compound **1**.

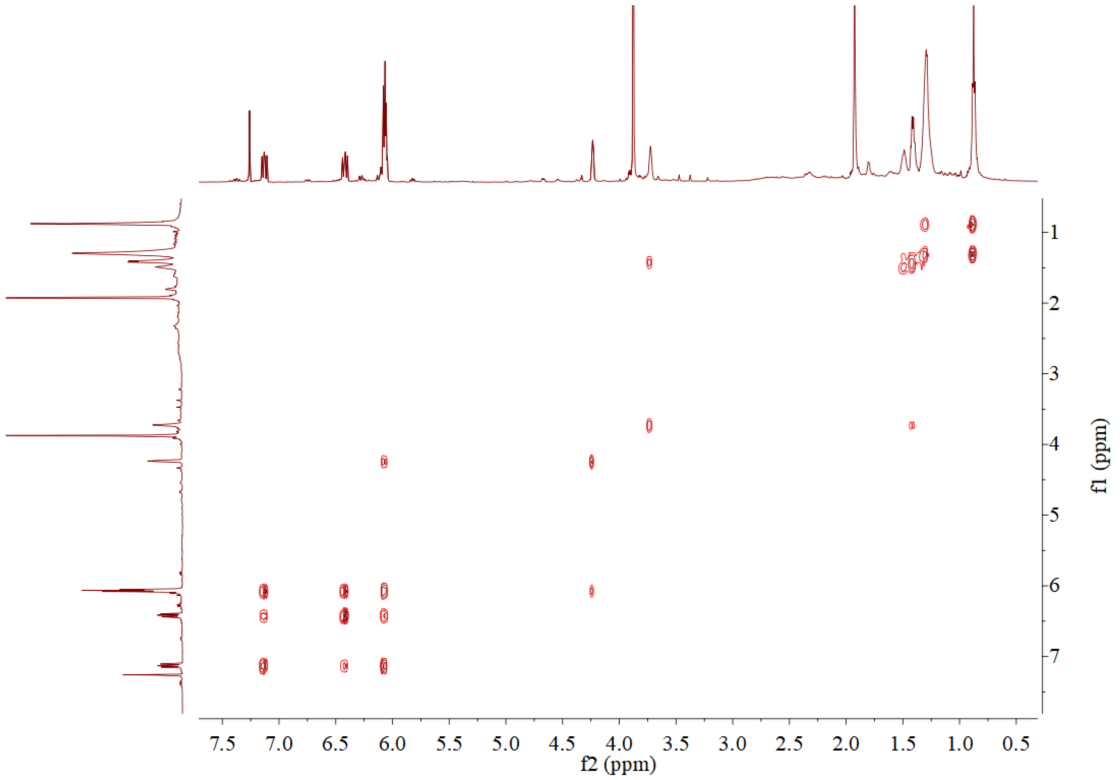

Figure S4. ^{1}H–^{1}H COSY ($CDCl_3$) spectrum of compound **1**.

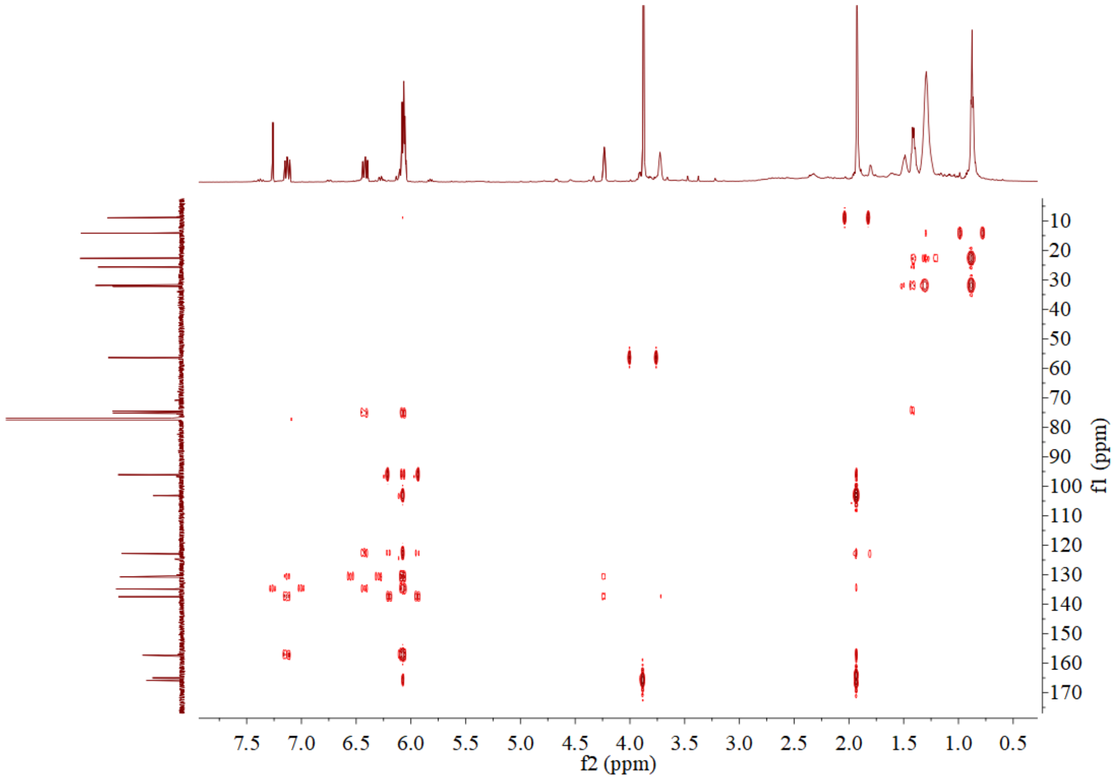

Figure S5. HMBC ($CDCl_3$) spectrum of compound **1**.

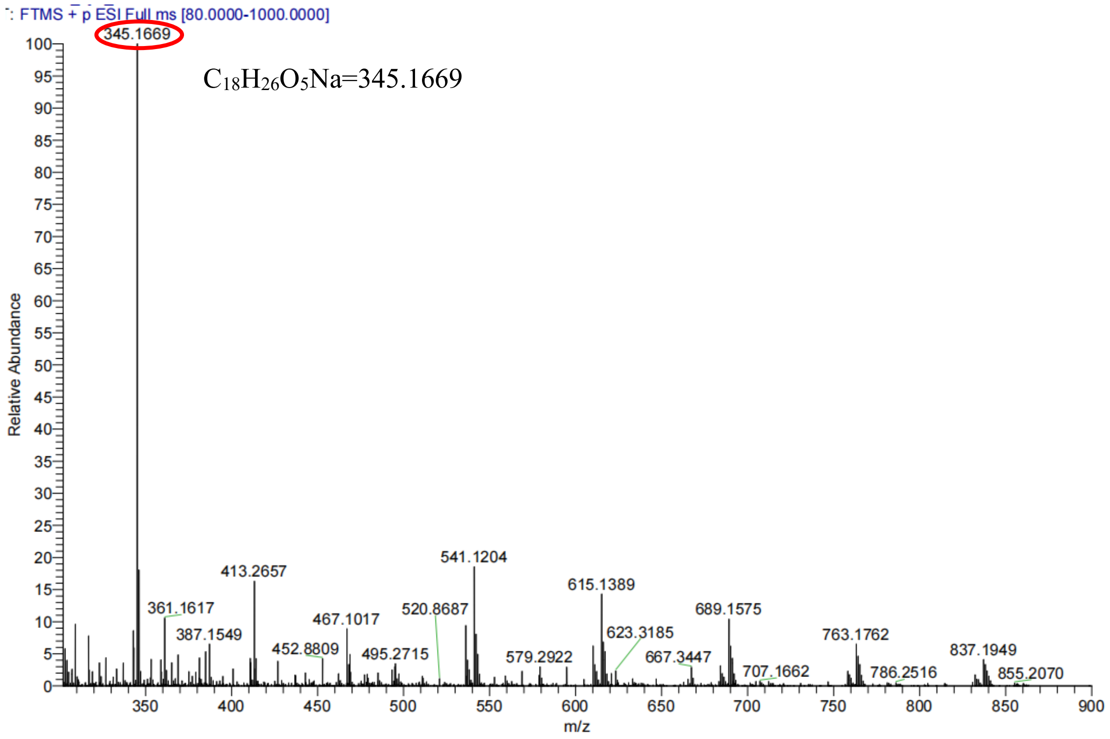

Figure S6. HRESIMS spectrum of compound **1**.

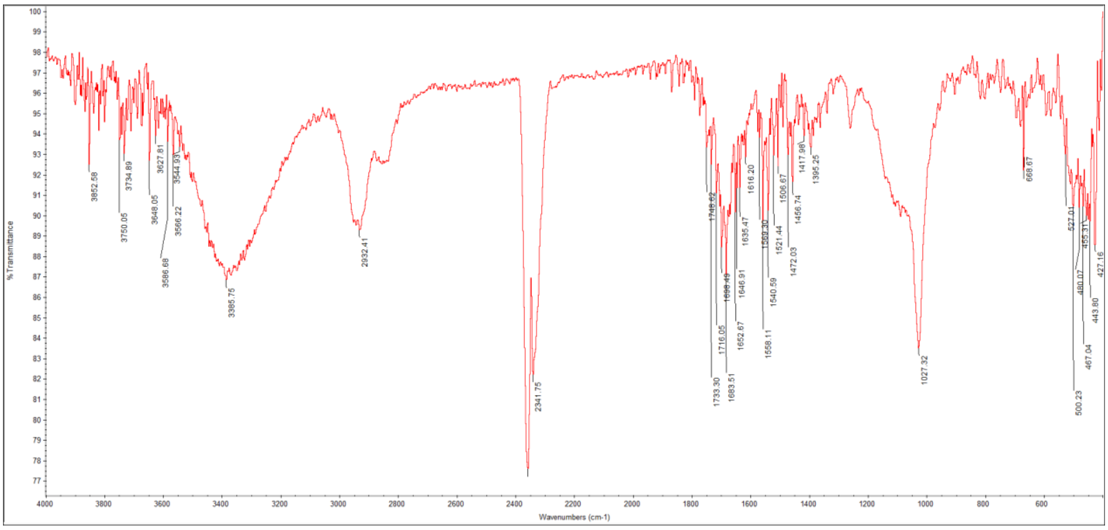

Figure S7. IR spectrum of compound **1.**

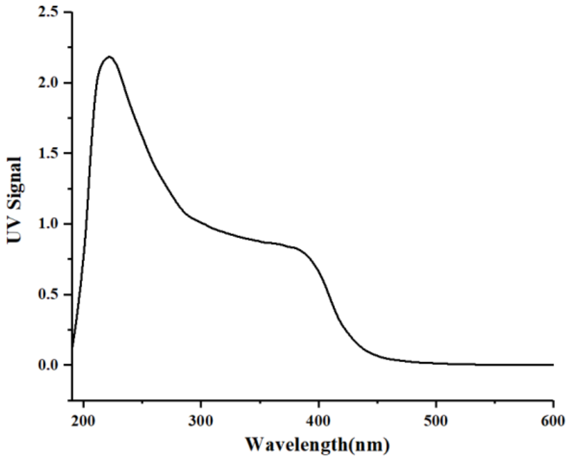

Figure S8. Experimental UV spectrum of **1**.

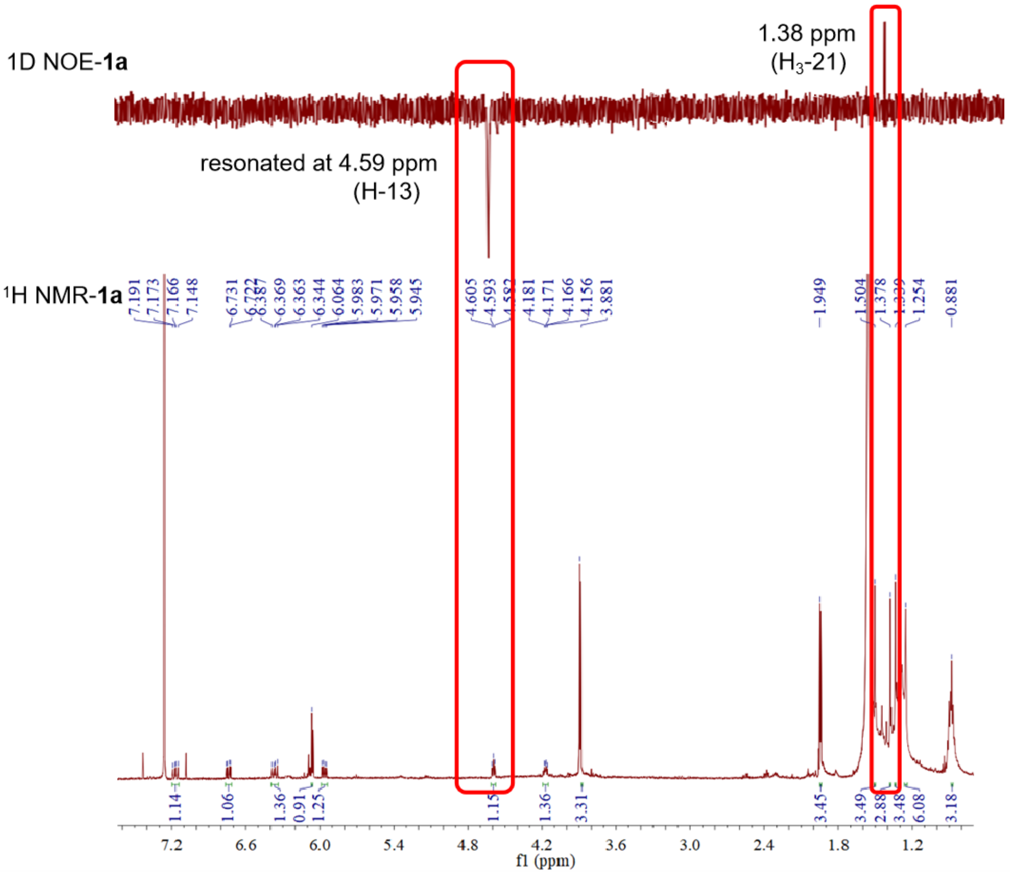

Figure S9. ^{1}H NMR and 1D NOE (resonated at H-13) spectra of compound **1a**.

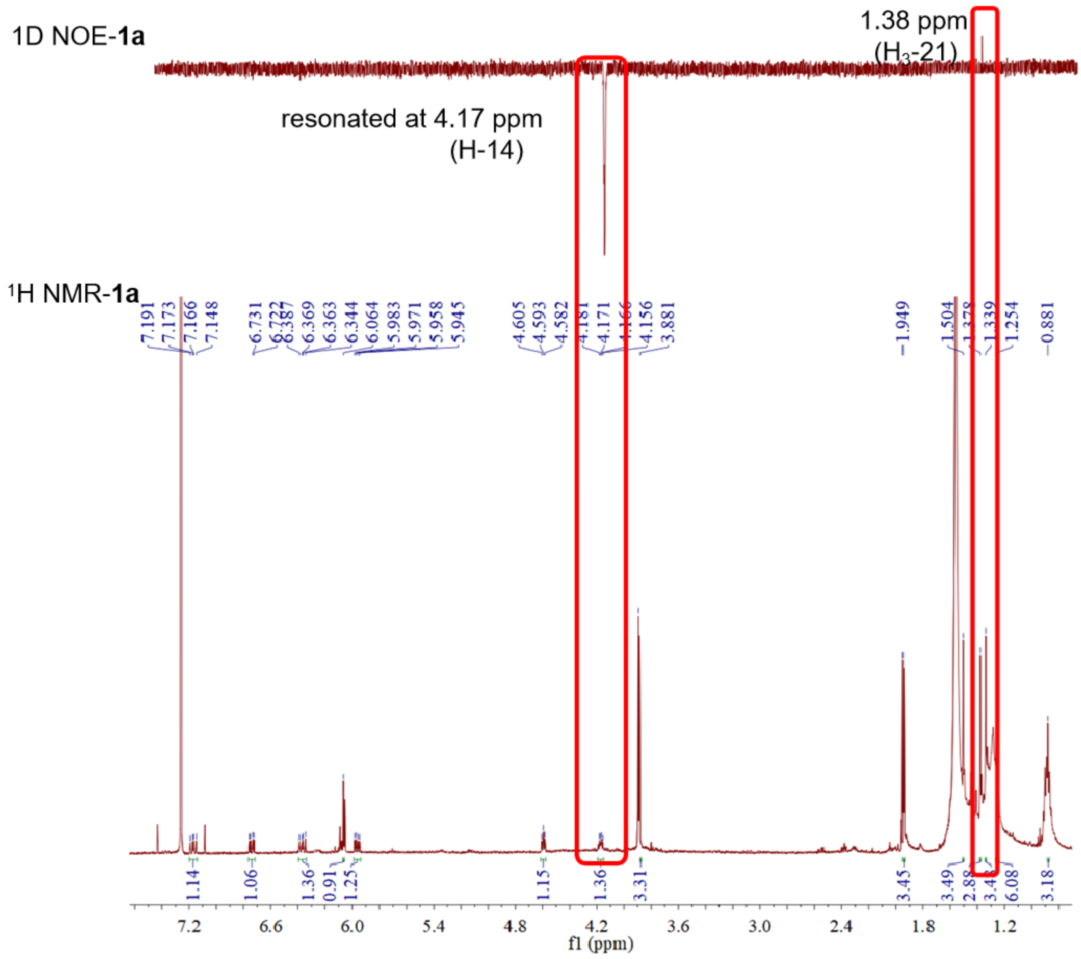

Figure S10. ^{1}H NMR and 1D NOE (resonated at H-14) spectra of compound **1a**.

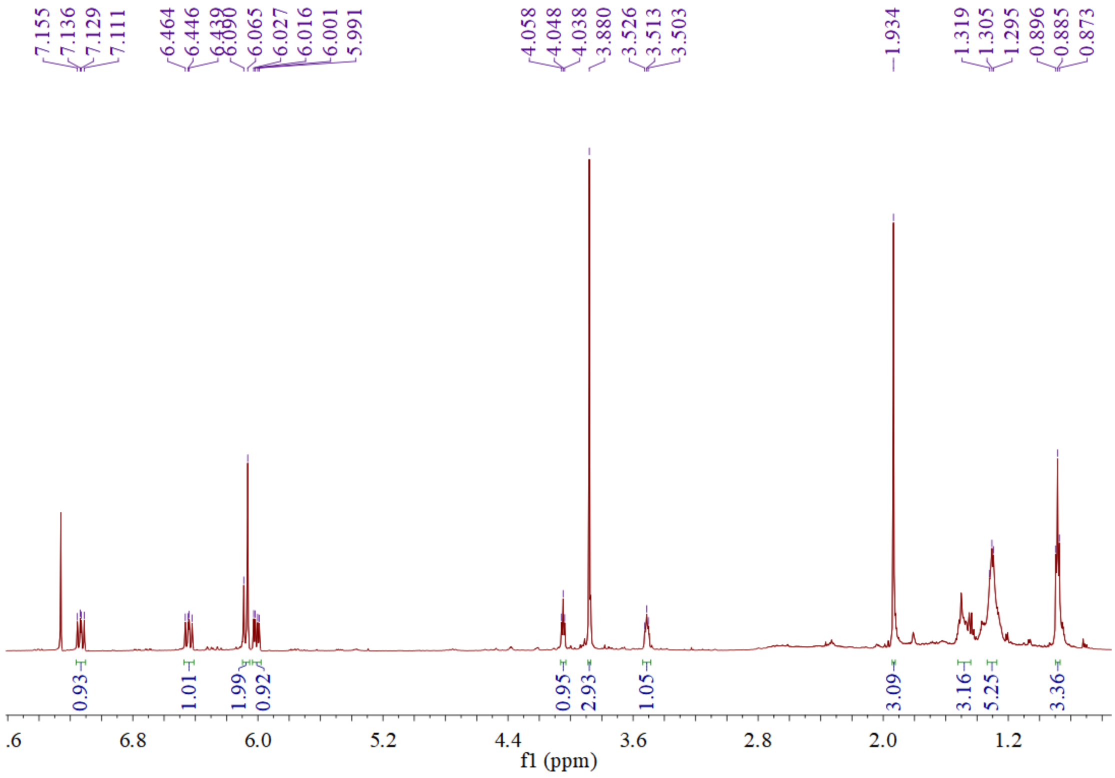

Figure S11. ^{1}H NMR (600 MHz, $CDCl_3$) spectrum of compound **2**.

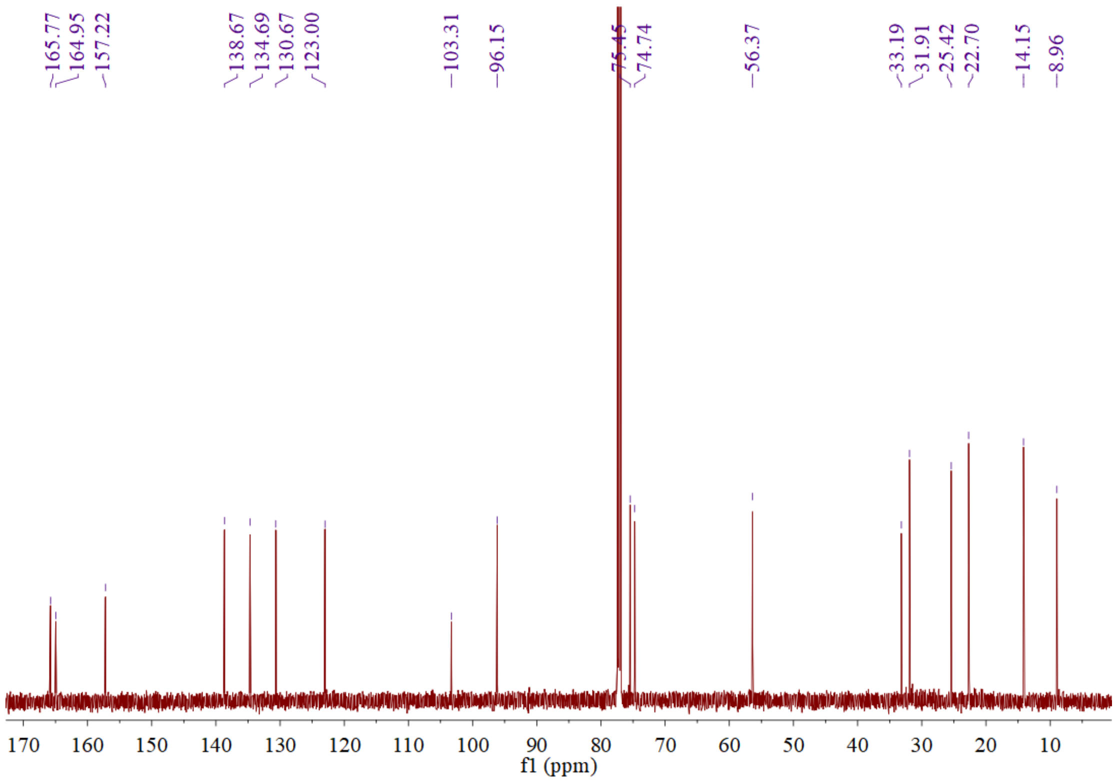

Figure S12. ^{13}C NMR (150 MHz, $CDCl_3$) spectrum of compound **2**.

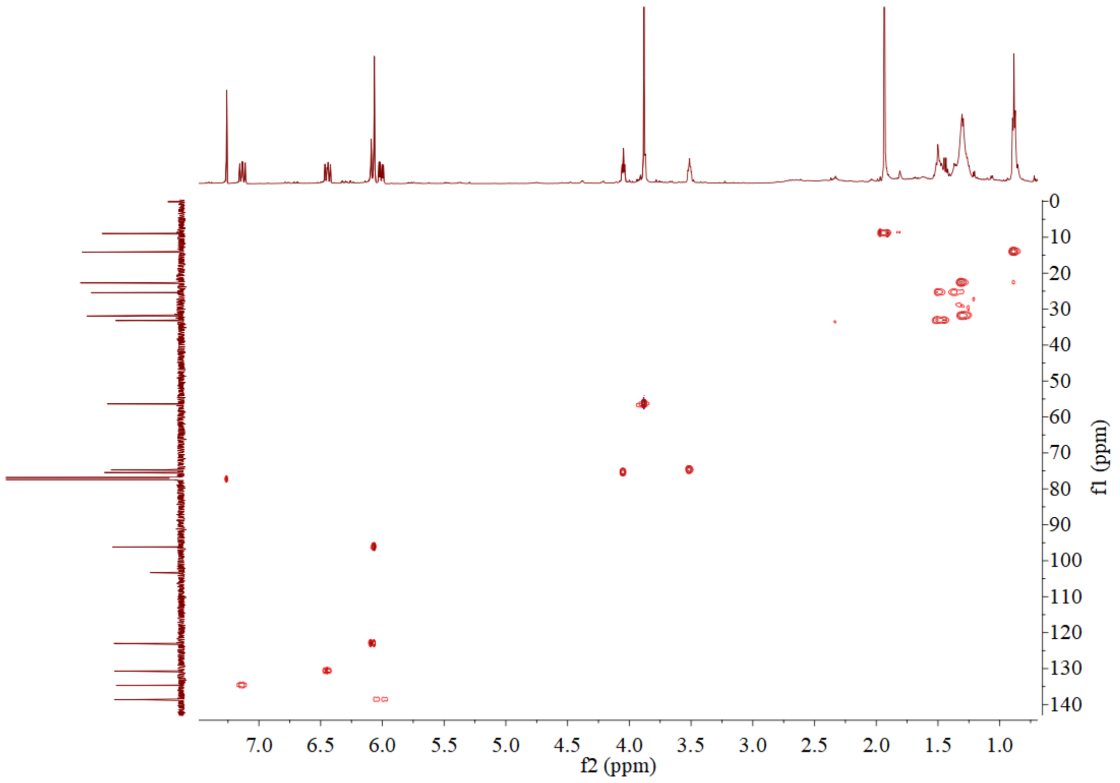

Figure S13. HSQC ($CDCl_3$) spectrum of compound **2**.

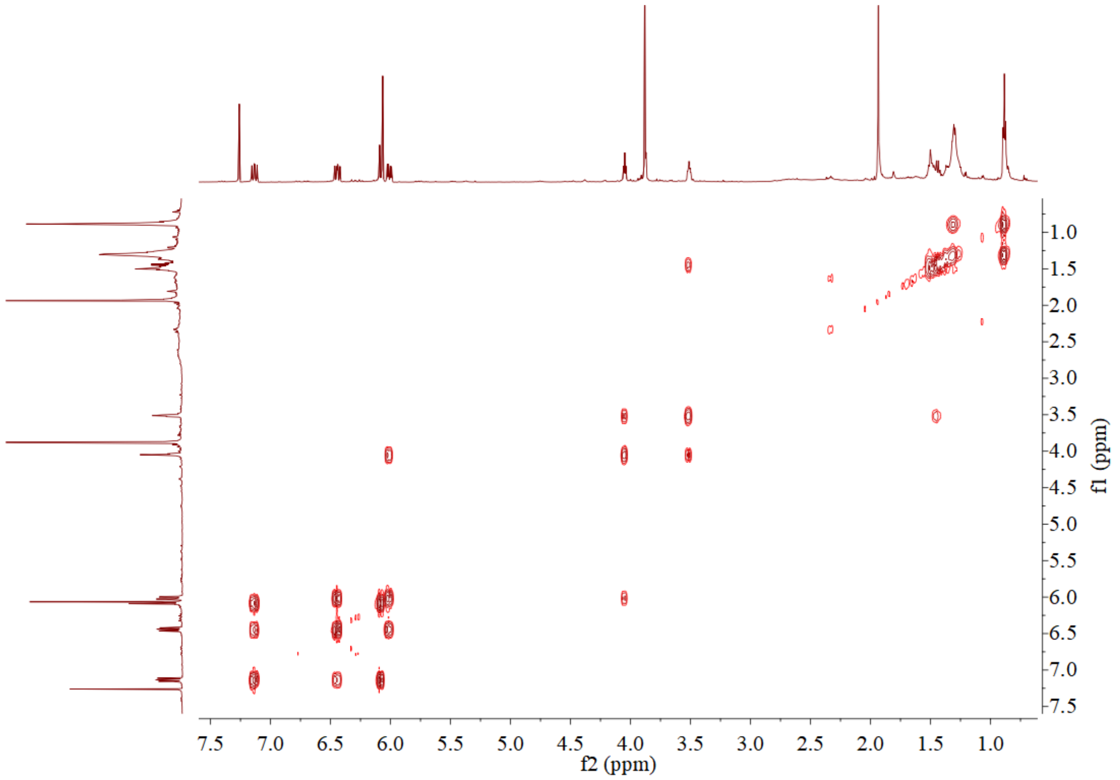

Figure S14. ^1H–^1H COSY ($CDCl_3$) spectrum of compound **2**.

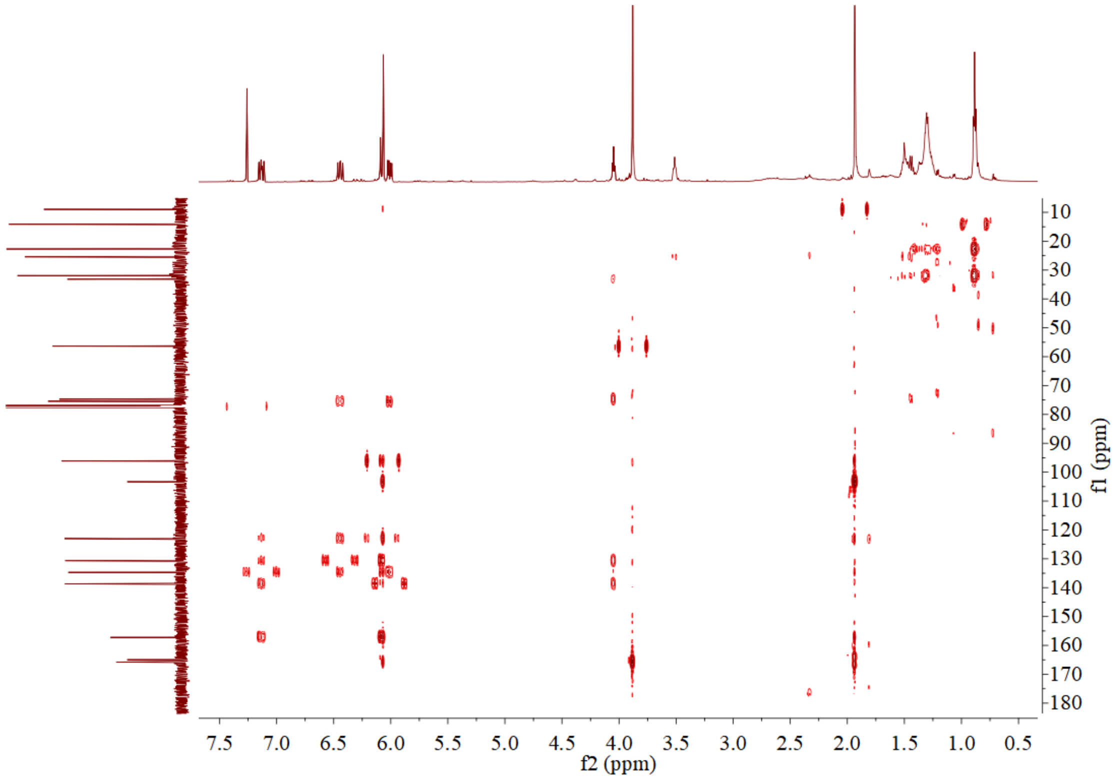

Figure S15. HMBC ($CDCl_3$) spectrum of compound **2**.

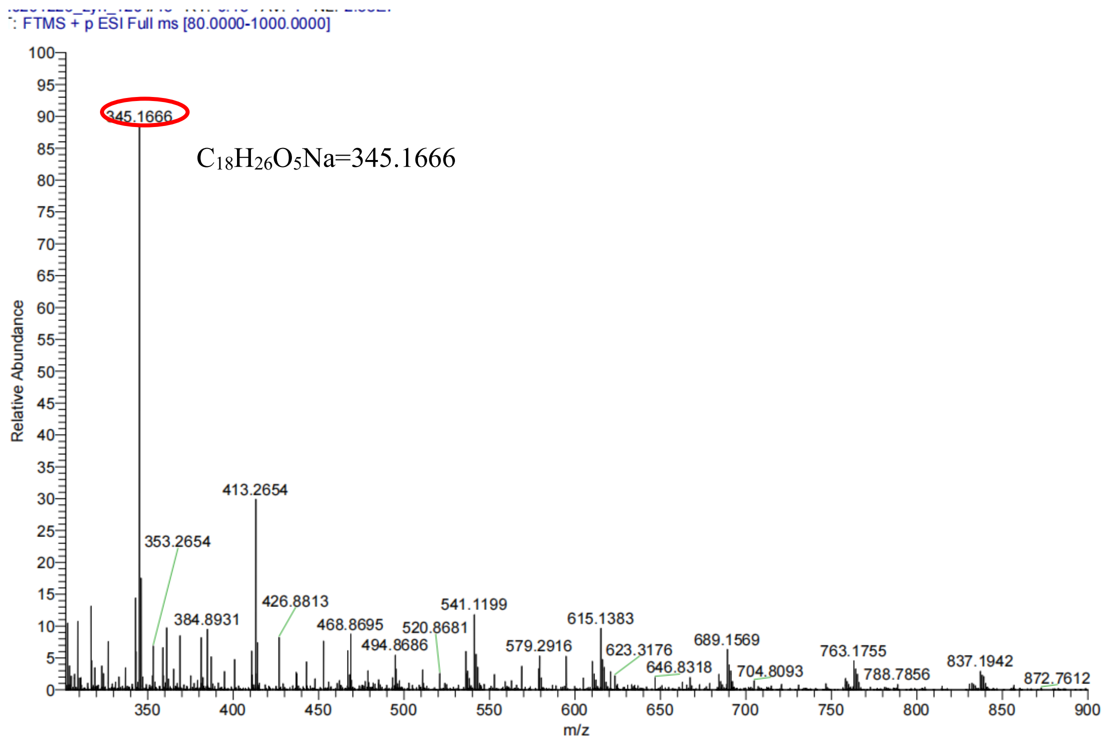

Figure S16. HRESIMS spectrum of compound **2**.

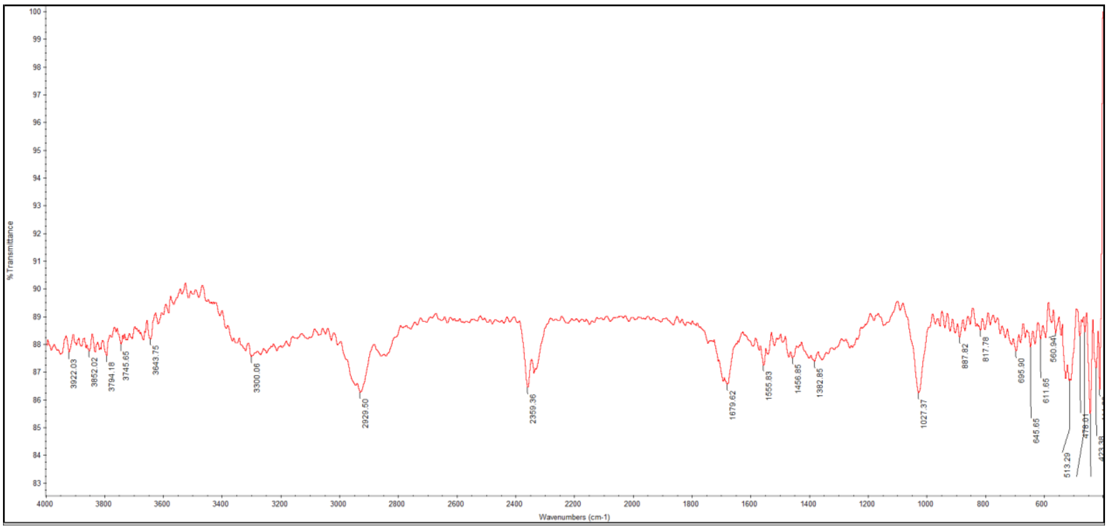

Figure S17. IR spectrum of compound **2.**

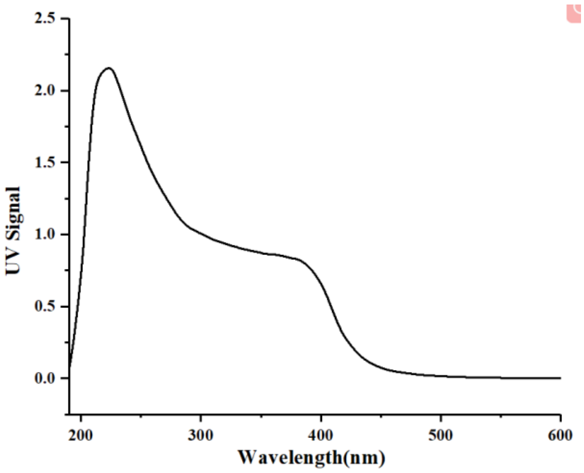

Figure S18. Experimental UV spectrum of **2**.

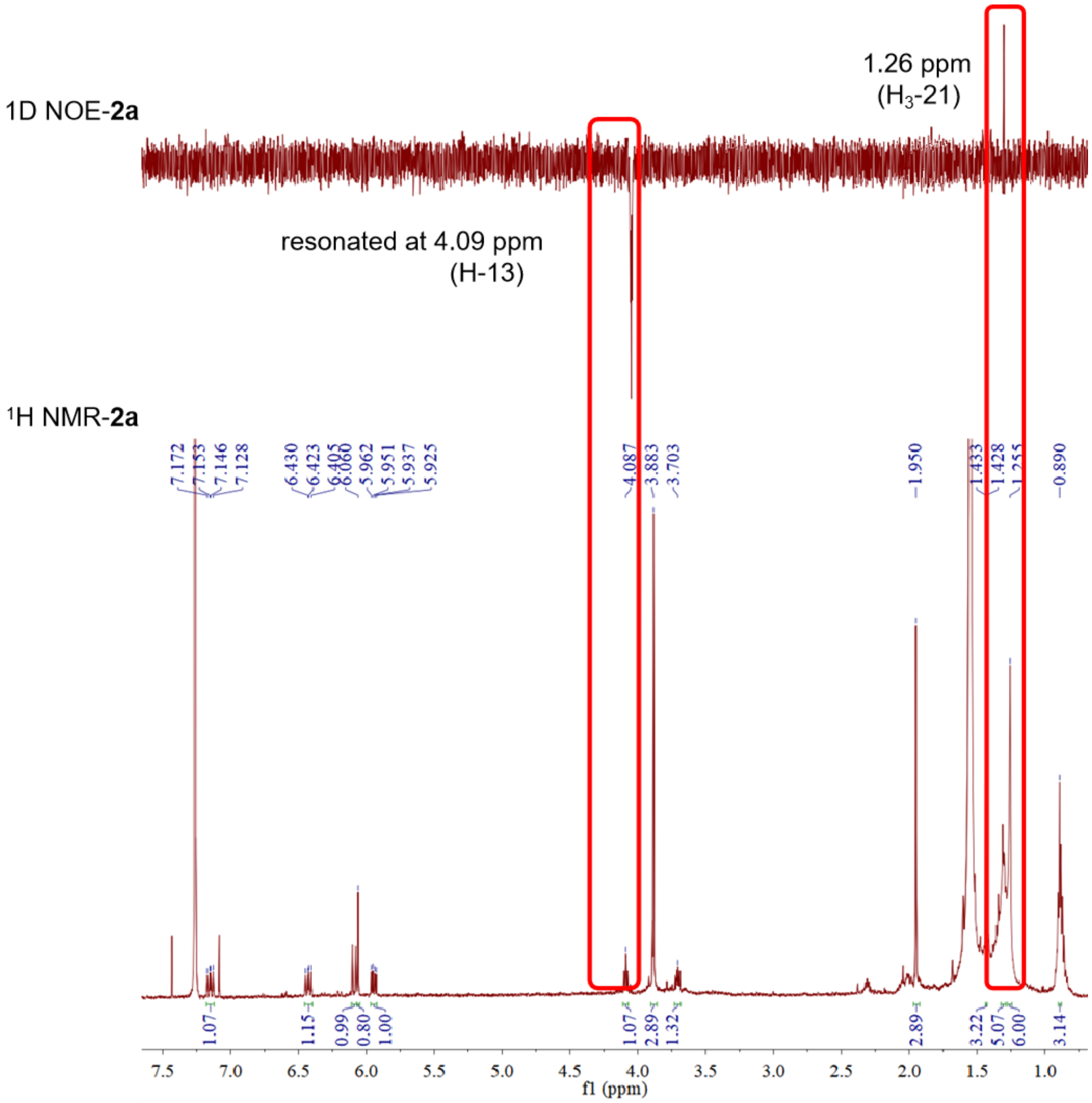

Figure S19. ^{1}H NMR and 1D NOE (resonated at H-13) spectra of compound **2a**

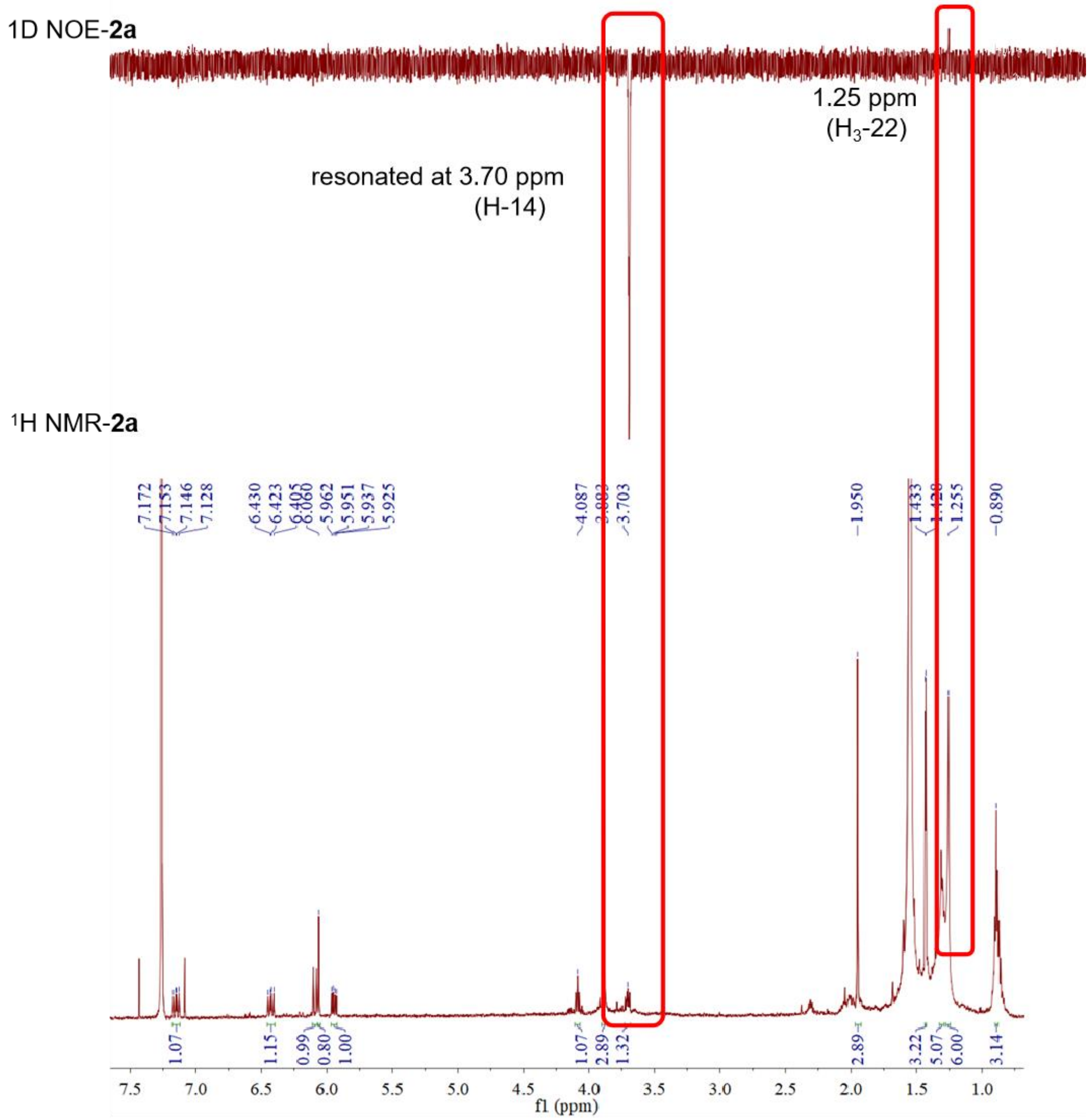

Figure S20. ^{1}H NMR and 1D NOE (resonated at H-14) spectra of compound **2a**

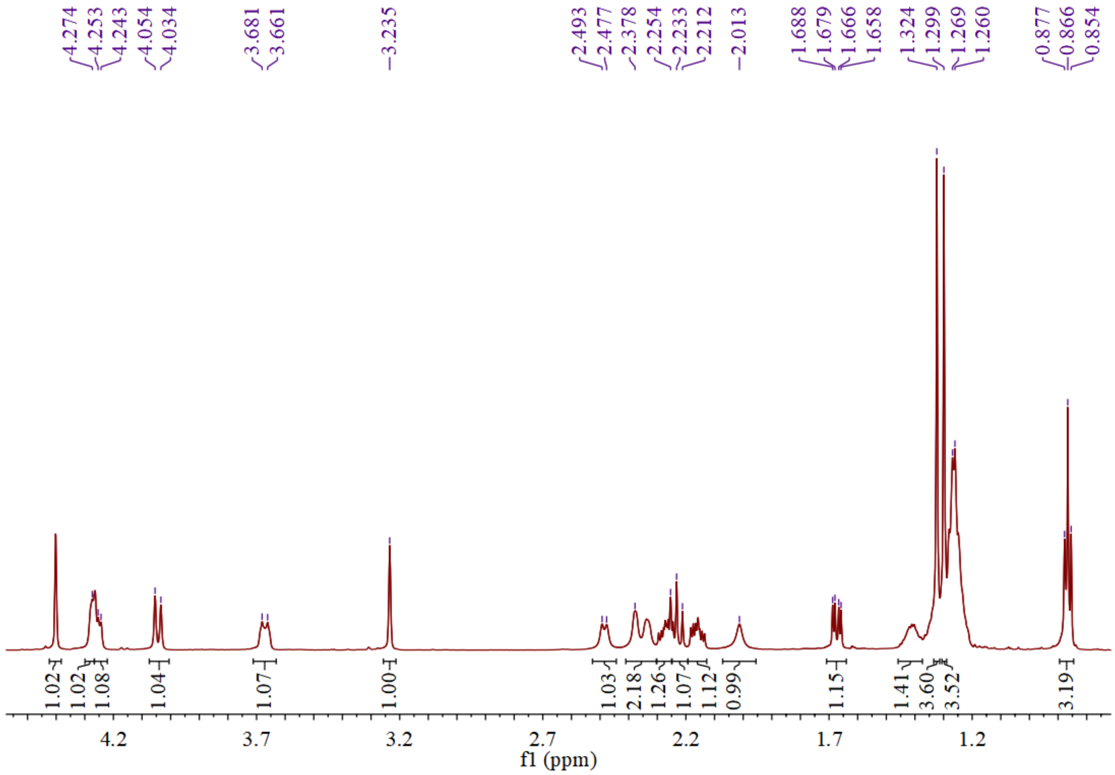

Figure S21. ^{1}H NMR (600 MHz, $CDCl_3$) spectrum of compound **3**.

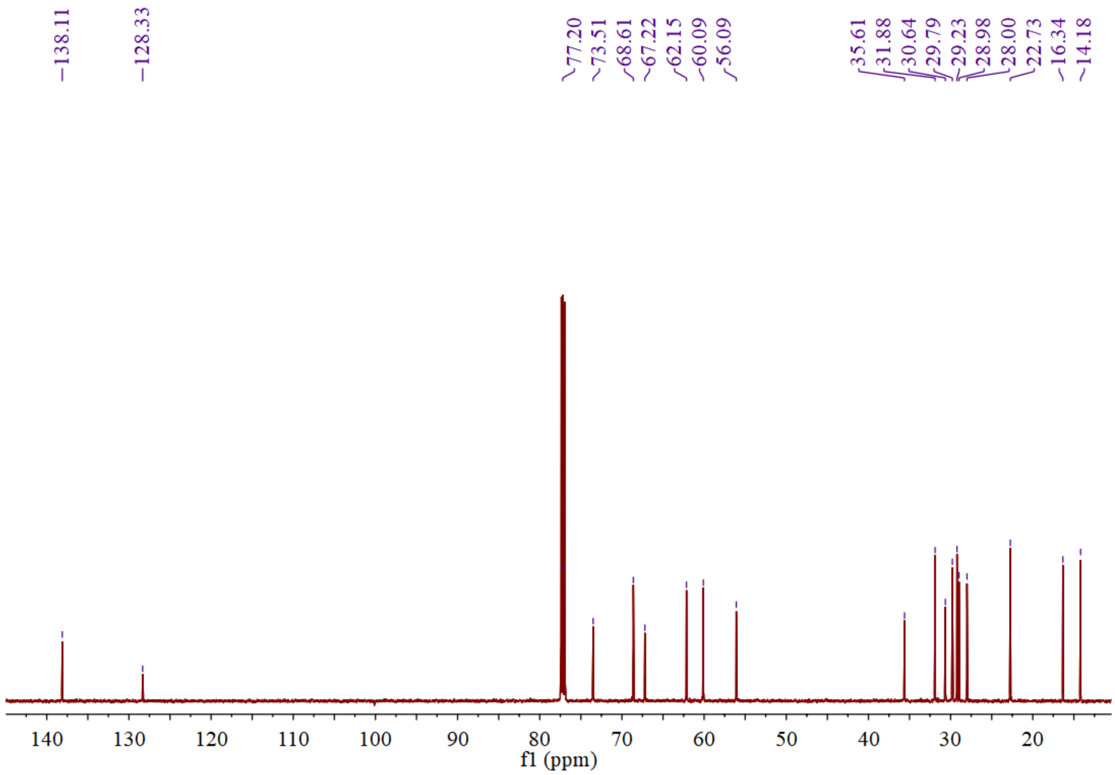

Figure S22. ^{13}C NMR (150 MHz, $CDCl_3$) spectrum of compound **3**.

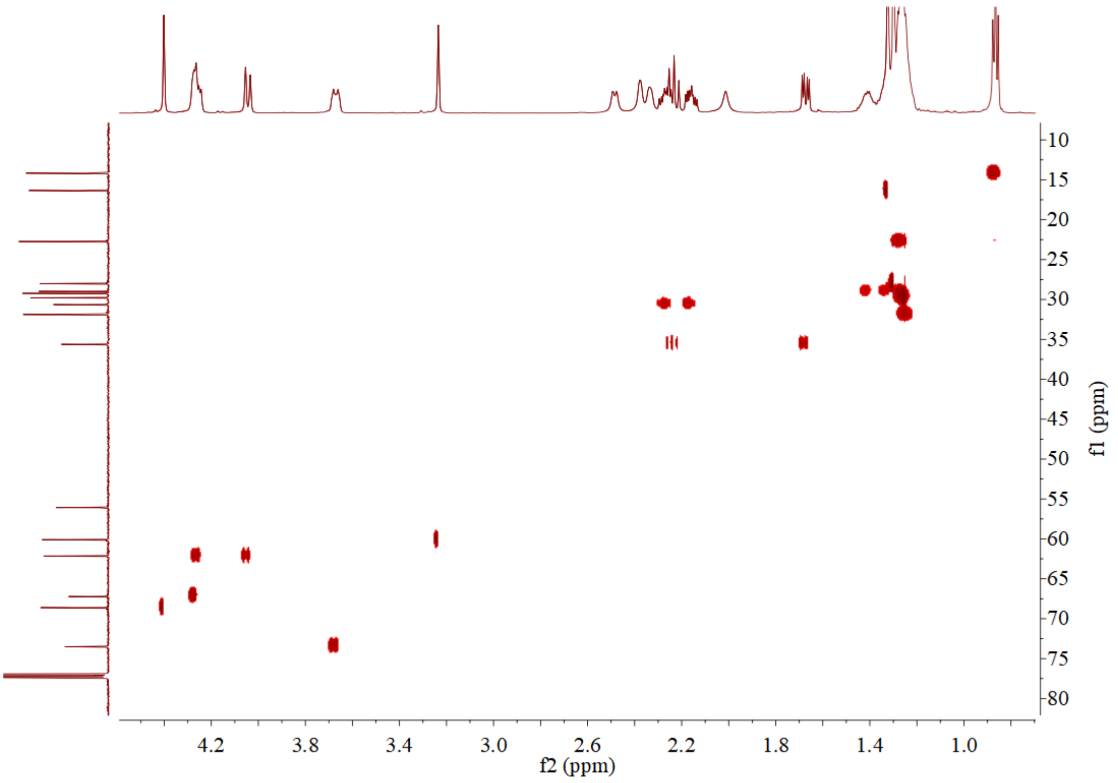

Figure S23. HSQC ($CDCl_3$) spectrum of compound **3**.

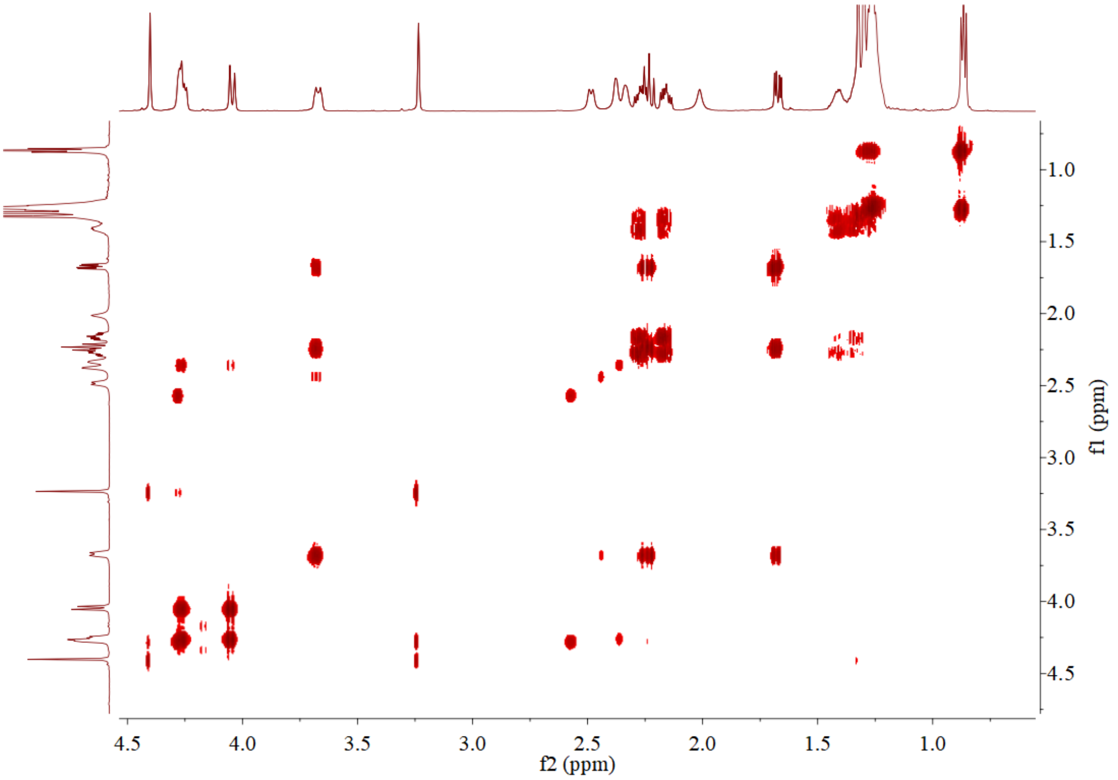

Figure S24. ^1H–^1H COSY ($CDCl_3$) spectrum of compound **3**.

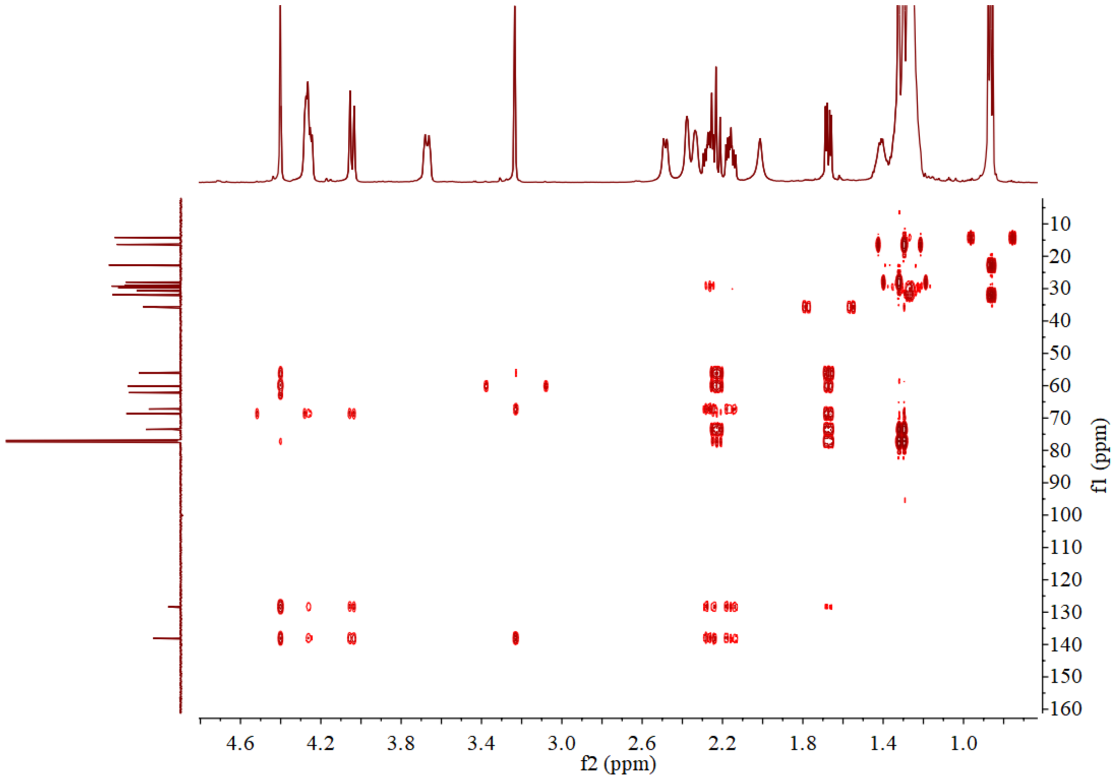

Figure S25. HMBC ($CDCl_3$) spectrum of compound **3**.

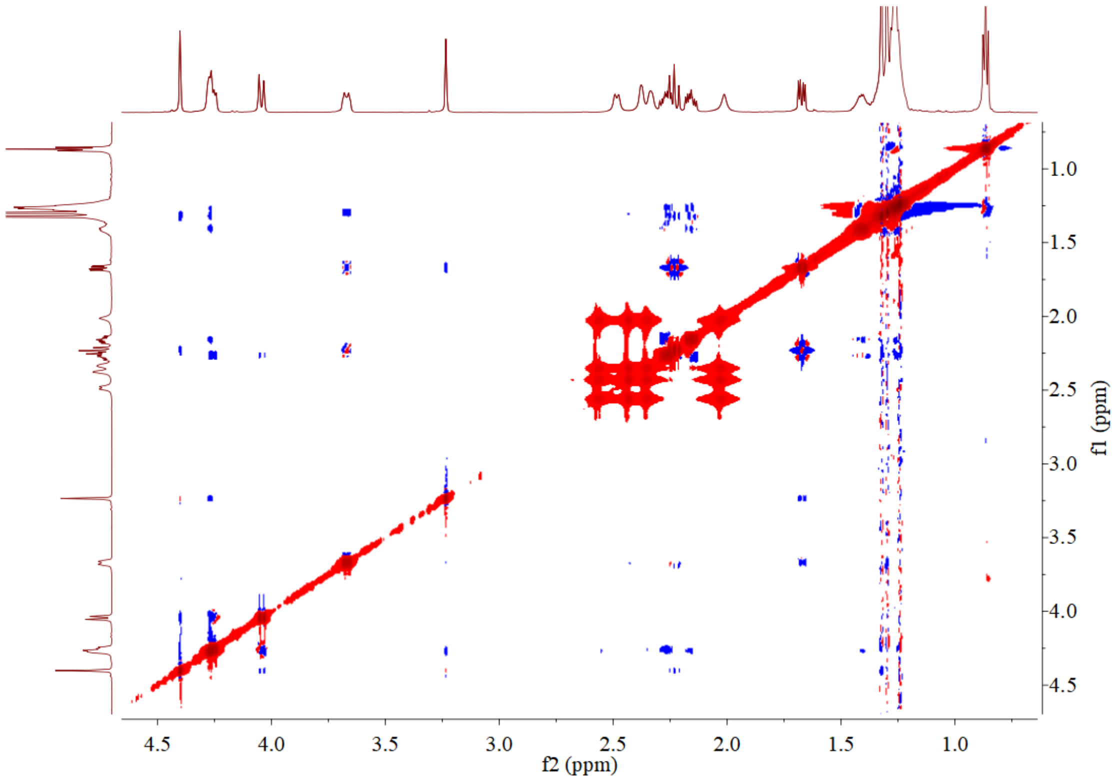

Figure S26. NOESY ($CDCl_3$) spectrum of compound **3**.

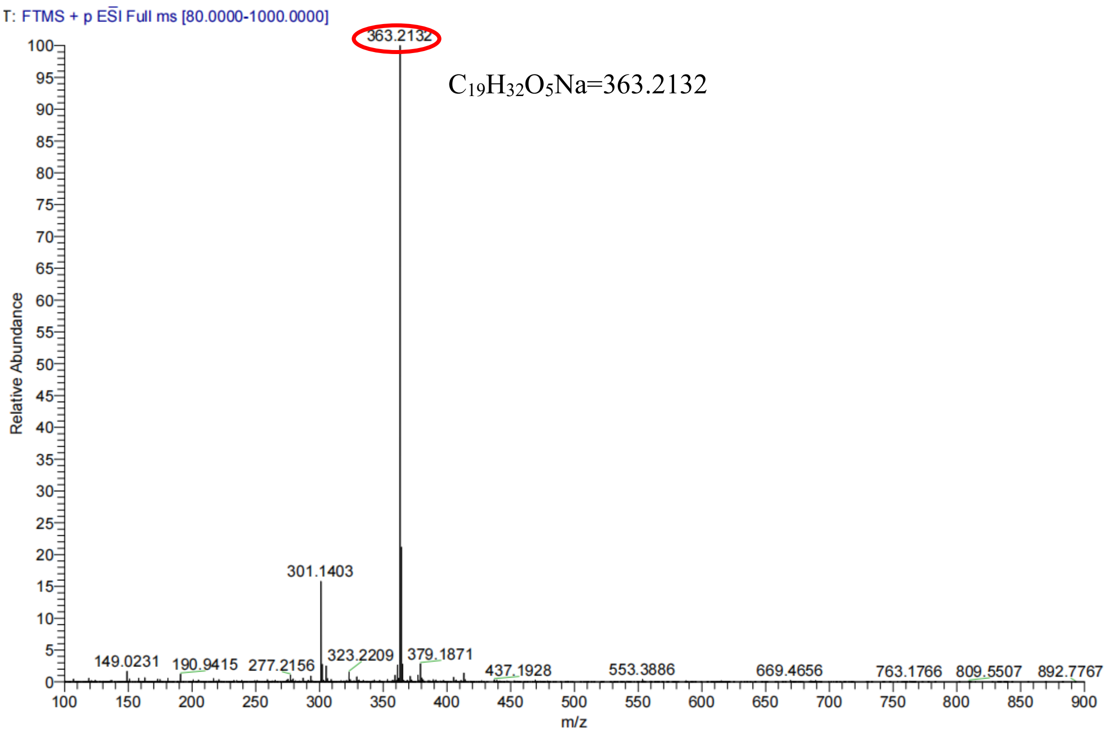

Figure S27. HRESIMS spectrum of compound **3**.

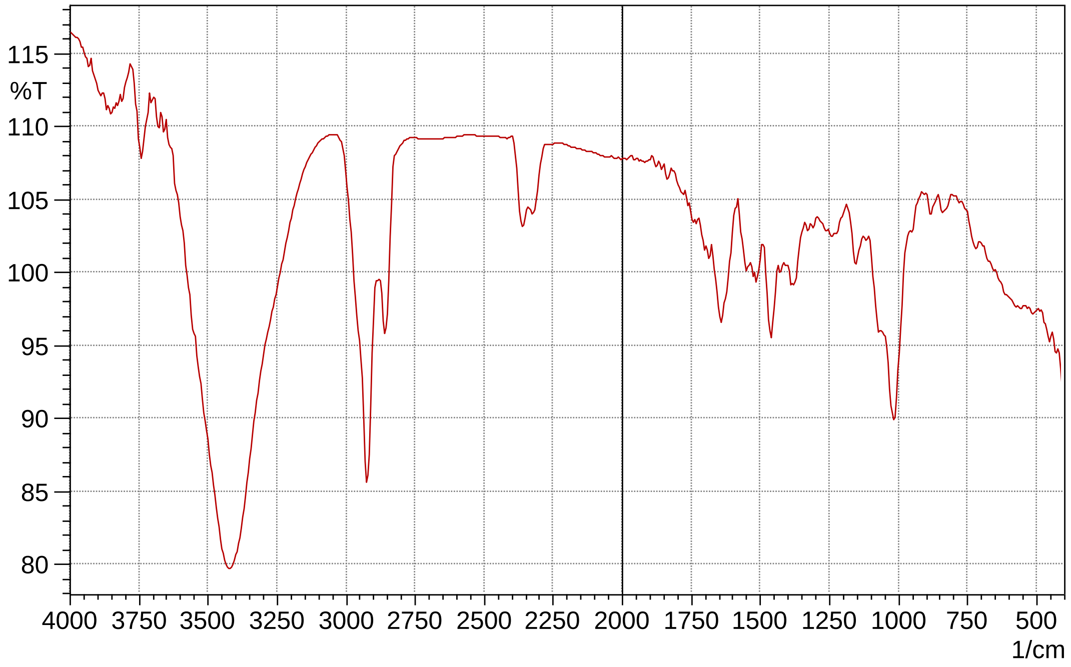

Figure S28. IR spectrum of compound **3.**

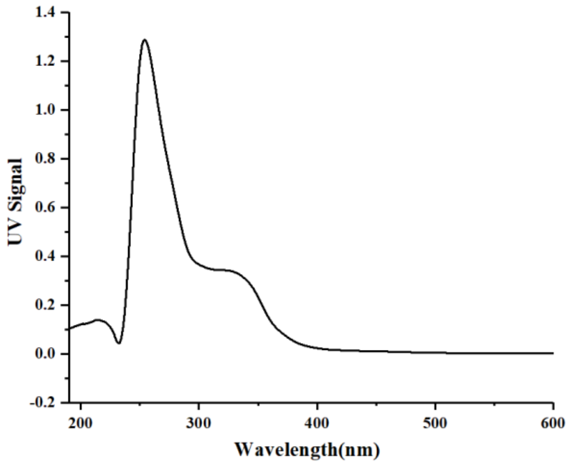

Figure S29. Experimental UV spectrum of **3**.

Table S1. Cytotoxic activity data of compounds **1**−**7**.

Concentration	Cell viability/%						
μg/mL	**1**	**2**	**3**	**4**	**5**	**6**	**7**
3.125	99.0	97.8	98.4	98.8	99.3	99.8	99.2
6.25	88.9	92.5	97.1	98.5	97.8	99.0	98.7
12.5	93.5	37.4	96.6	98.1	93.5	98.2	97.8
25.0	93.9	9.2	96.0	92.8	89.4	96.1	96.4
50.0	45.2	6.4	92.7	82.7	88.8	93.3	95.9

Table S2. Anti-inflammatory activity data of compounds **1**−**7**.

Concentration	NO Production inhibition ratio/%						
μg/mL	**1**	**2**	**3**	**4**	**5**	**6**	**7**
0.78	-	-0.9	-	-	-	-	-
1.56	-	4.2	-	-	-	-	-
3.125	8.0	14.4	7.2	5.9	5.1	10.1	0.4
6.25	20.3	1.7	-1.7	5.1	24.1	13.5	-4.6
12.5	11.8	-	4.2	3.8	27.4	21.1	2.1
25.0	13.1	-	8.0	6.3	32.1	35.9	6.3
50.0	-	-	4.2	0.4	49.0	54.9	3.4

Table S3. The coordinate for the lowest-energy conformer of (13*S*,14*S*)-**1b** in ECD calculation

	Coordinates (Angstroms)		
	X	Y	Z
C	4.50287800	0.80584100	0.20991200
C	4.34954800	-0.55855100	0.09492800
C	3.05565900	-1.13418000	-0.10708100
C	1.96565000	-0.31324900	-0.18659400
O	2.09394500	1.02605400	-0.07932500
C	3.34334000	1.66312900	0.12333700
C	5.82385200	1.48880800	0.42529600
O	5.46977200	-1.32000500	0.18221000
C	5.37152500	-2.73482000	0.06721300
O	3.32656800	2.87115000	0.20517100
C	0.61272300	-0.78615600	-0.38611600
C	-0.47585400	0.01454500	-0.46565100
C	-1.82125400	-0.47609000	-0.66485600
C	-2.90951500	0.31546700	-0.74151900
C	-4.30680200	-0.17896700	-0.95739300
C	-5.29239600	0.19157000	0.17035200
C	-4.97000100	-0.48457800	1.50072900
C	-5.93995600	-0.08557300	2.61734200
O	-4.87357700	0.43567700	-2.13259400
O	-6.60068800	-0.20087600	-0.21894200
H	2.91244800	-2.20208800	-0.19706000
H	6.02171300	2.20704000	-0.37916800
H	5.81199400	2.06588400	1.35769700
H	6.64071000	0.76690400	0.46595100
H	4.75217400	-3.16267200	0.86495700
H	4.97212100	-3.03345400	-0.90968300
H	6.39216000	-3.10737800	0.16665300
H	0.50281200	-1.86549600	-0.47128400
H	-0.34050500	1.09018300	-0.37557200
H	-1.94519300	-1.55662900	-0.75470700
H	-2.79778800	1.39732300	-0.66158900
H	-4.31309800	-1.27456300	-1.06675500
H	-5.24657000	1.28852300	0.29703300
H	-5.00273900	-1.57245200	1.35165900
H	-3.94164200	-0.23023500	1.78590600
H	-6.96809600	-0.34099000	2.34627900
H	-5.69102100	-0.59881000	3.55280100
H	-5.90019100	0.99408900	2.80748400

H	-4.34072700	0.16688400	-2.89672400
H	-6.70310300	0.10875700	-1.13542600

Table S4. The coordinate for the lowest-energy conformer of (13*R*,14*R*)-**1b** in ECD calculation

	Coordinates (Angstroms)		
	X	Y	Z
C	-4.50286600	0.80599600	0.20968900
C	-4.34958900	-0.55846000	0.09539700
C	-3.05573000	-1.13424500	-0.10636300
C	-1.96568200	-0.31340200	-0.18628600
O	-2.09392200	1.02596000	-0.07969000
C	-3.34329800	1.66319700	0.12262900
C	-5.82382600	1.48913100	0.42462000
O	-5.46982300	-1.31984000	0.18326800
C	-5.37178700	-2.73462500	0.06765700
O	-3.32648000	2.87126600	0.20373400
C	-0.61277500	-0.78646800	-0.38556900
C	0.47583200	0.01415600	-0.46548800
C	1.82122000	-0.47660400	-0.66444700
C	2.90948100	0.31490900	-0.74158300
C	4.30676200	-0.17964200	-0.95727300
C	5.29244900	0.19167600	0.17013200
C	4.97026200	-0.48368800	1.50096400
C	5.94022900	-0.08380800	2.61725200
O	4.87338900	0.43423800	-2.13294400
O	6.60075100	-0.20086800	-0.21904500
H	-2.91254900	-2.20221700	-0.19563300
H	-6.64070900	0.76727800	0.46568600
H	-5.81200700	2.06679500	1.35665400
H	-6.02161600	2.20686400	-0.38031100
H	-4.75309000	-3.16296100	0.86565700
H	-6.39256300	-3.10703300	0.16621600
H	-4.97173800	-3.03288600	-0.90907900
H	-0.50290900	-1.86585300	-0.47020200
H	0.34051200	1.08984100	-0.37592600
H	1.94515500	-1.55719300	-0.75370100
H	2.79775700	1.39680700	-0.66223800
H	4.31306500	-1.27530900	-1.06591900
H	5.24653200	1.28870000	0.29614700
H	3.94187400	-0.22937500	1.78606300
H	5.00320800	-1.57164300	1.35254600
H	6.96839800	-0.33919300	2.34626200
H	5.90027000	0.99596400	2.80672600
H	5.69147600	-0.59650900	3.55305200

H	4.34045800	0.16493500	-2.89683900
H	6.70306800	0.10821200	-1.13572700

Table S5. The coordinate for the lowest-energy conformer of (13*R*,14*S*)-**1b** in ECD calculation

	Coordinates (Angstroms)		
	X	Y	Z
C	-4.40910700	0.90478100	0.09732800
C	-4.32695200	-0.46830900	0.17316500
C	-3.06508600	-1.13243100	0.05955700
C	-1.93359100	-0.38754200	-0.12313100
O	-1.99133100	0.95873400	-0.19779300
C	-3.20618100	1.68123300	-0.09563000
C	-5.69312800	1.67792300	0.20385000
O	-5.48537300	-1.15157700	0.35751600
C	-5.46040100	-2.57135000	0.44691200
O	-3.12677300	2.88651700	-0.18011100
C	-0.60780200	-0.95329900	-0.25097300
C	0.52342000	-0.22903900	-0.41836700
C	1.83932800	-0.81497700	-0.54894100
C	2.97121100	-0.10057800	-0.70666200
C	4.33275400	-0.70682700	-0.87054900
C	5.39461400	-0.10303600	0.07093800
C	5.06008700	-0.25832500	1.55178300
C	6.18047800	0.24397300	2.46768300
O	4.84644300	-0.43137000	-2.19114600
O	5.54979900	1.28110200	-0.21505700
H	-2.97841800	-2.20873700	0.11477800
H	-5.65673000	2.37212100	1.05202500
H	-5.84706900	2.29254900	-0.69092400
H	-6.54741000	1.01103800	0.32838800
H	-6.49866200	-2.87282800	0.59378100
H	-5.07972900	-3.02772400	-0.47501000
H	-4.86204700	-2.91150700	1.30091000
H	-0.55710800	-2.03935600	-0.20295000
H	0.44931000	0.85549600	-0.45857600
H	1.89971900	-1.90434900	-0.51305100
H	2.93304500	0.98728200	-0.73842600
H	4.28369100	-1.79491100	-0.70729000
H	6.33630300	-0.63810100	-0.14481300
H	4.13246500	0.28850000	1.76159300
H	4.85544100	-1.31915600	1.75232600
H	5.90426700	0.13230700	3.52198800
H	7.10859300	-0.31737800	2.30305000
H	6.38889600	1.30048600	2.27660100

H	4.12614700	-0.57779200	-2.82408900
H	5.66930200	1.33161100	-1.17935300

Table S6. The coordinate for the lowest-energy conformer of (13*S*,14*R*)-**1b** in ECD calculation

	Coordinates (Angstroms)		
	X	Y	Z
C	4.40913000	0.90476600	0.09735300
C	4.32696800	-0.46833500	0.17307100
C	3.06510300	-1.13244300	0.05941000
C	1.93360500	-0.38753100	-0.12317400
O	1.99134600	0.95875300	-0.19766800
C	3.20620100	1.68124100	-0.09548300
C	5.69316200	1.67788200	0.20391600
O	5.48538900	-1.15163700	0.35727100
C	5.46032600	-2.57138800	0.44697400
O	3.12678600	2.88653700	-0.17979500
C	0.60781600	-0.95327500	-0.25107000
C	-0.52340600	-0.22899400	-0.41838200
C	-1.83931500	-0.81491200	-0.54901900
C	-2.97119400	-0.10048500	-0.70664100
C	-4.33273700	-0.70670800	-0.87062500
C	-5.39461700	-0.10298600	0.07088700
C	-5.06022600	-0.25858500	1.55172800
C	-6.18064200	0.24364800	2.46763300
O	-4.84639000	-0.43112600	-2.19120600
O	-5.54967000	1.28121800	-0.21486500
H	2.97844200	-2.20875900	0.11446600
H	5.84705200	2.29264800	-0.69077000
H	5.65682900	2.37194300	1.05220600
H	6.54744600	1.01096800	0.32829200
H	6.49854900	-2.87288000	0.59407700
H	4.86181700	-2.91134100	1.30094300
H	5.07978400	-3.02793600	-0.47491900
H	0.55712100	-2.03933700	-0.20316200
H	-0.44929200	0.85554500	-0.45846600
H	-1.89971200	-1.90428900	-0.51326400
H	-2.93302300	0.98737800	-0.73826700
H	-4.28369000	-1.79480900	-0.70746400
H	-6.33632900	-0.63794200	-0.14503700
H	-4.85569100	-1.31947300	1.75208800
H	-4.13256900	0.28811300	1.76171100
H	-5.90450300	0.13179900	3.52193600
H	-6.38897700	1.30020500	2.27670400
H	-7.10878500	-0.31761500	2.30285600

H	-4.12607300	-0.57747400	-2.82414300
H	-5.66913100	1.33193100	-1.17915500

Table S7. The coordinate for the lowest-energy conformer of (3*S*,5*R*,6*S*,7*R*,10*S*)-**3a** in ECD calculation

	Coordinates (Angstroms)		
	X	Y	Z
C	-2.66645100	-0.81023100	0.31480100
C	-2.42592700	0.71275600	0.19183100
O	-0.99134800	0.92527000	0.30527900
C	-0.13741800	0.27364000	-0.66824200
C	-0.36918300	-1.22336300	-0.62247800
C	-1.82911600	-1.61173900	-0.68855800
C	1.27144300	0.73851400	-0.37348900
C	2.20636700	-0.05465000	0.17306600
C	1.90247800	-1.47807800	0.59661600
C	0.65431800	-2.07449700	-0.00903500
C	3.62546200	0.38316400	0.47198700
O	0.52134900	-2.00098400	-1.43895800
O	1.80560300	-1.55908700	2.03030800
O	-4.06422600	-1.04252800	0.13921700
C	-3.01658900	1.44097300	1.39645200
C	-2.98280800	1.30221500	-1.10814400
C	1.48838500	2.20536600	-0.67994700
O	0.98103200	3.05245900	0.35467600
H	-0.41145000	0.63072600	-1.67117300
H	-2.36957800	-1.09708100	1.33320600
H	-1.93605900	-2.68533400	-0.50000000
H	-2.20341000	-1.43019100	-1.70160600
H	2.75193900	-2.12424400	0.35155900
H	0.36242000	-3.03145200	0.42063600
H	3.65781300	1.44545300	0.72083400
H	3.96357400	-0.14515700	1.36876500
H	1.22113600	-0.85145300	2.33483700
H	-4.26243100	-1.96752800	0.32698200
H	-2.55959700	1.08473200	2.32272200
H	-2.83859100	2.51627300	1.31986800
H	-4.09253600	1.26975500	1.45085600
H	-4.06849900	1.20116100	-1.12560200
H	-2.73087900	2.36376200	-1.16729200
H	-2.59881500	0.81286000	-2.00468800
H	2.54306500	2.45454900	-0.78074800
H	1.00584900	2.45239900	-1.63697600
H	0.11315900	2.70675200	0.60957000
C	4.59482300	0.08827600	-0.68594000

H	5.61856600	0.35642800	-0.41045600
H	4.59290900	-0.97150100	-0.95659200
H	4.32743500	0.65398400	-1.58239500

Table S8. The coordinate for the lowest-energy conformer of compound (3*R*,5*S*,6*R*,7*S*,10*R*)-**3a** for ECD calculation

	Coordinates (Angstroms)		
	X	Y	Z
C	2.66645100	-0.81023100	0.31480100
C	2.42592700	0.71275600	0.19183100
O	0.99134800	0.92527000	0.30527800
C	0.13741800	0.27364000	-0.66824200
C	0.36918400	-1.22336300	-0.62247800
C	1.82911700	-1.61173900	-0.68855800
C	-1.27144300	0.73851400	-0.37348900
C	-2.20636700	-0.05465000	0.17306600
C	-1.90247900	-1.47807800	0.59661600
C	-0.65431800	-2.07449700	-0.00903500
C	-3.62546200	0.38316400	0.47198700
O	-1.80560300	-1.55908700	2.03030800
O	4.06422600	-1.04252800	0.13921700
C	2.98280800	1.30221400	-1.10814400
C	3.01658900	1.44097300	1.39645200
C	-1.48838500	2.20536600	-0.67994700
O	-0.98103300	3.05245900	0.35467700
H	0.41145000	0.63072600	-1.67117300
H	2.36957800	-1.09708100	1.33320600
H	2.20341000	-1.43019200	-1.70160600
H	1.93605900	-2.68533400	-0.49999900
H	-2.75193900	-2.12424400	0.35155900
H	-3.96357400	-0.14515700	1.36876500
H	-3.65781200	1.44545300	0.72083300
H	-1.22113700	-0.85145300	2.33483700
H	4.26243100	-1.96752700	0.32698200
H	4.06849900	1.20116000	-1.12560300
H	2.59881500	0.81285900	-2.00468800
H	2.73087900	2.36376200	-1.16729200
H	2.55959700	1.08473300	2.32272200
H	4.09253600	1.26975600	1.45085500
H	2.83859100	2.51627300	1.31986700
H	-1.00584800	2.45239900	-1.63697600
H	-2.54306500	2.45454900	-0.78074800
H	-0.11315900	2.70675200	0.60957000
C	-4.59482300	0.08827600	-0.68594100
H	-5.61856600	0.35642800	-0.41045600

H	-4.32743400	0.65398400	-1.58239500
H	-4.59290900	-0.97150100	-0.95659200
H	-0.36242000	-3.03145200	0.42063600
O	-0.52134900	-2.00098400	-1.43895800
